# Geographic encoding of transcripts enabled high-accuracy and isoform-aware deep learning of RNA methylation

**DOI:** 10.1093/nar/gkac830

**Published:** 2022-09-26

**Authors:** Daiyun Huang, Kunqi Chen, Bowen Song, Zhen Wei, Jionglong Su, Frans Coenen, João Pedro de Magalhães, Daniel J Rigden, Jia Meng

**Affiliations:** Department of Biological Sciences, Xi'an Jiaotong-Liverpool University, Suzhou 215123, PR China; Department of Computer Sciences, University of Liverpool, Liverpool L69 7ZB, UK; Key Laboratory of Gastrointestinal Cancer (Fujian Medical University), Ministry of Education, School of Basic Medical Sciences, Fujian Medical University, Fuzhou 350004, PR China; Department of Mathematical Sciences, Xi'an Jiaotong-Liverpool University, Suzhou 215123, PR China; Institute of Systems, Molecular and Integrative Biology, University of Liverpool, Liverpool L69 7ZB, UK; Department of Biological Sciences, Xi'an Jiaotong-Liverpool University, Suzhou 215123, PR China; Institute of Life Course and Medical Sciences, University of Liverpool, Liverpool L69 7ZB, UK; Department of Mathematical Sciences, Xi'an Jiaotong-Liverpool University, Suzhou 215123, PR China; School of AI and Advanced Computing, Xi'an Jiaotong-Liverpool University, Suzhou 215123, PR China; Department of Computer Sciences, University of Liverpool, Liverpool L69 7ZB, UK; Institute of Life Course and Medical Sciences, University of Liverpool, Liverpool L69 7ZB, UK; Institute of Systems, Molecular and Integrative Biology, University of Liverpool, Liverpool L69 7ZB, UK; Department of Biological Sciences, Xi'an Jiaotong-Liverpool University, Suzhou 215123, PR China; Institute of Systems, Molecular and Integrative Biology, University of Liverpool, Liverpool L69 7ZB, UK; AI University Research Centre, Xi’an Jiaotong-Liverpool University, Suzhou 215123, PR China

## Abstract

As the most pervasive epigenetic mark present on mRNA and lncRNA, *N^6^*-methyladenosine (m^6^A) RNA methylation regulates all stages of RNA life in various biological processes and disease mechanisms. Computational methods for deciphering RNA modification have achieved great success in recent years; nevertheless, their potential remains underexploited. One reason for this is that existing models usually consider only the sequence of transcripts, ignoring the various regions (or geography) of transcripts such as 3′UTR and intron, where the epigenetic mark forms and functions. Here, we developed three simple yet powerful encoding schemes for transcripts to capture the submolecular geographic information of RNA, which is largely independent from sequences. We show that m^6^A prediction models based on geographic information alone can achieve comparable performances to classic sequence-based methods. Importantly, geographic information substantially enhances the accuracy of sequence-based models, enables isoform- and tissue-specific prediction of m^6^A sites, and improves m^6^A signal detection from direct RNA sequencing data. The geographic encoding schemes we developed have exhibited strong interpretability, and are applicable to not only m^6^A but also *N^1^*-methyladenosine (m^1^A), and can serve as a general and effective complement to the widely used sequence encoding schemes in deep learning applications concerning RNA transcripts.

## INTRODUCTION

Post-transcriptional RNA modifications expand RNA molecule's functional and structural diversity (1) and regulate its metabolism at all stages of RNA life (2–5). More than 100 different post-transcriptional RNA modifications have been identified in all three kingdoms of life (6). Among them, *N*^6^-methyladenosine (m^6^A) is the most common modification in eukaryotic mRNA and lncRNA (7). m^6^A occurs on nascent pre-mRNA (8), modulating its translation (9,10) and is involved in many essential biological processes, such as differentiation from naïve pluripotency (11,12), circadian clock (13), and the heat shock response (14). It also plays various roles in disease development and mechanisms, such as breast tumor (15), gastric cancer (16), carcinoma (17) and anti-tumor immunity (18). Therefore, the precise identification of modification sites is of crucial importance for understanding the functional and regulatory circuitry of RNA.

Thanks to advances in high-throughput sequencing, a number of experimental approaches have been developed to profile the entire epitranscriptome (19). Among them, MeRIP-seq (or m^6^A-seq) (20,21) is the first method to detect transcriptome-wide m^6^A RNA methylation, and technically can be viewed as a marriage of RNA-seq and ChIP-seq, where the fragmented RNAs are first immunoprecipitated by anti-m^6^A antibody, and then purified and sequenced for the detection of m^6^A signals.

Since experimental approaches for studying RNA modification are expensive and laborious, *in silico* methods have drawn increasing attention as an alternative avenue and have achieved great success in recent years. To date, more than 100 different approaches (22–26) have been established for computational prediction of RNA modification sites, including most notably, the iRNA toolkit (27–36), SRAMP (37), WHISTLE (38), Gene2vec (39), PEA (40), DeepPromise (25), MASS (41), m6Aboost (42), MultiRM (43), DeepAc4C (44), WeakRM (45), PULSE (46), NmRF (47), etc. Among them, the iRNA toolkit (27–36) developed primarily by Chen, Lin and Chou is the earliest as well as the most versatile toolkit, supporting multiple RNA modification types based on RNA primary sequences and has been widely recognized as the gold standard for benchmarking the accuracy of different RNA modification prediction approaches. By taking advantage of various state-of-the-art machine learning and deep learning techniques, the previous work has greatly advanced our understanding of the localization and sequence characteristics of multiple RNA modifications under various biological conditions and in different organisms.

Existing approaches for RNA modification site prediction are mostly based on the primary sequences only. This is not surprising given that the primary sequences of DNA, RNA, and protein convey the most fundamental information of the biomolecules and have been predominantly used as the primary information source for existing machine learning tools in biosciences. There exist a large number of sequence-based methods to address various life science challenges, such as the prediction of biological functions and structures (48–51). Meanwhile, many tools have been developed to facilitate feature extraction and machine learning modeling of the primary sequences, such as bioSeq-Analysis (52), PyFeat (53) and PseKRAAC (54). These tools have achieved enormous success, especially for obtaining insights under biological contexts not adequately explored by wet-experimental approaches. However, limited by the computational resources available to handle large datasets and the capability of deep learning models, in many cases only a local fraction rather than the entire transcript is used for prediction tasks, and a substantial amount of information is therefore discarded in the process. Although the distant sequences discarded from the analysis could, in theory, contain useful information as well, that information can not be effectively extracted with current machine learning models. In the problem of RNA modification site prediction (55), conventional machine learning algorithms typically consider only a local RNA fragment of 20–50-nt (29,32,33) when predicting whether a specific ribonucleotide is modifiable or not. Even though some of the latest deep learning approaches may take advantage of up to 2000-nt flanking sequences of the target, that may still represent a relatively small fragment of the entire RNA molecule that can be millions of nucleotides long. Not being able to take advantage of information related to the entire RNA molecule may limit the potential of *in silico* approaches.

On a separate note, to supplement sequence information, transcript annotation has been used as another information source for predicting RNA modifications. This is natural because both the transcript structure and the relative position on the transcript are found to be related to the occurrence and function of RNA sub-molecular events. For example, the most prevalent RNA modification, *N*^6^-methyladenosine (m^6^A), is enriched on the last long exons and 3′UTRs (20,21,56), and can affect alternative splicing (57); the microRNA target sites bounded by Argonaute (AGO) proteins were shown to be predominantly located in the CDS and 3′UTR of the target mRNAs but not 5′UTRs (58). Therefore, encoding the sub-region information (geography) of the transcript may be useful for deep learning models applied to RNA transcripts. We previously developed WHISTLE (38) as a high-accuracy m^6^A site predictor, which incorporated 35 genomic features besides the conventional sequence features, including the transcript region information, such as the region type (3′UTR, CDS, 5′UTR, etc.) of the target ribonucleotide or whether it is within an exon with a width >400-nt. Although only 41-nt long RNA sequences were seen in the WHISTLE method, its performance is comparable to the state-of-the-art deep learning models based on thousands of nucleotides of input sequences. Recently, miCLIP2 (42) also considered region type information in their machine learning model. In the RNA binding protein (RBP) target prediction problem, transcript information has been represented as one-hot encoded region type features (59,60), e.g. each 50-nt upstream and downstream relative to the RBP binding site was assigned into five types of transcript region: exons, intron, CDS, 5′UTR and 3′UTR, resulting in 101 × 5 region type features. More recently, DeepRiPe (61), a deep learning approach for predicting and interpreting RBP target sites, also used these region-type indicators and considered a 250-nt window as a suitable range. However, these approaches may suffer from the following limitations. Although the region features defined in WHISTLE enabled significant improvement in the prediction performance, they cannot effectively capture the relative positional information with respect to the long-range region boundaries, e.g. exon/intron junctions and stop codons. Additionally, 35 genomic features were independently defined, and a uniform logic is unavailable for the automatic extension of the framework to other more general regional annotations. The widely used one-hot encoding of the region type features within a fixed-length window typically results in an incomplete landscape of the local transcript structure. Furthermore, the region type feature matrix contains lots of redundant information in the form of consecutive identical vectors, suggesting that this encoding scheme is still crude. In general, it is still an open question how best to extract the geographic information of ribonucleotides with respect to the functional sub-regions of the entire RNA transcript.

In this study, we explored different strategies for encoding sub-molecular geographic information of ribonucleotides and developed a tool called **geo**graphic representation of transcript as **vec**tors (Geo2vec), which implements three novel encoding methods, landmarkTX, gridTX and chunkTX, as well as the widely used one-hot method. LandmarkTX is a lightweight encoding scheme directly capturing the position of the target ribonucleotide (or site) relative to transcript landmarks, e.g. the distances to the two edges of the exon, coding sequence (CDS), and transcript, respectively. Meanwhile, gridTX and chunkTX are designed to describe the landscape of the entire transcript through grids (of equal widths) or regions (with unequal width), respectively. The novelty of the newly proposed Geo2vec method relates to the following three aspects. First, compared to the local contextual information captured by existing one-hot encoded region type features, Geo2vec retains more transcript structure with lighter weight (landmarkTX has only 6 features, and chunkTX has only 245 features). Geo2vec not only captures the complete landscape of transcripts but also makes the model aware of the relationship between the target site (ribonucleotide of interest) and the region boundaries. Second, although the transcript encoding constructed by Geo2vec is at a single transcript level, it allows us to deal with isoform ambiguity of RNA by encoding each isoform transcript as a separate feature matrix and then pooling all the isoforms together in the deep neural networks. Third, recent advances in deep learning model interpretation methods allow us to explore the contribution of each input feature after obtaining a well-trained neural network model. The interpretation of Geo2vec descriptors can provide biological insights into the relationship between the target and the transcript landscape. Together, Geo2vec provides general, lightweight, more informative, and interpretable sub-molecular geographic descriptors of transcripts, which are largely independent from the widely used sequence descriptors.

Using m^6^A site prediction as a test case, we evaluated the effects of different geographic encoding schemes. Our results suggested that the performance of the m^6^A prediction model based on geographic information alone (AUC of 0.807) is already comparable to the classic sequence-based approaches such as MethyRNA (AUC of 0.790) (62), and incorporating additional geographic information can substantially enhance the accuracy of the state-of-the-art sequence-based learning model DeepPromise (25), with 3.2% higher AUC score and a 3.3% higher improvement in AP (Average Precision) score. Additionally, we explored the impact of isoform ambiguity on m^6^A site prediction and developed an attention-based multiple instance learning framework to fully use the isoform transcript information. By combining our previously developed WeakRM framework (45) and Geo2vec, we constructed isoform-aware high-accuracy tissue-specific m^6^A predictors for 25 human tissues (with mean AUC of 0.893 and mean AP 0.873). Compared with the sequence-only model, the AUC is 8% higher, and the AP is 10.7% higher, showing the importance of distinguishing isoform-specific methylation. Furthermore, the interpretation analysis indicated that the m^6^A is enriched within long exons and the 3′-end exons, which is consistent with existing knowledge. We also demonstrated its usage in constructing a technically robust m^6^A site predictor and detecting m^6^A signals from Oxford Nanopore direct RNA sequencing data. Overall, Geo2vec will be a useful tool for submolecular geographic encoding of transcripts, providing additional complementary information that is largely independent from their sequences and delivering novel biological insights owing to its strong interpretability.

## MATERIALS AND METHODS

### Raw data and preprocessing

Four sets of reported m^6^A sites were used in base-resolution m^6^A prediction. The first two datasets, denoted as the sramp17 benchmark dataset and sramp17 independent testing dataset, were constructed by mapping the coordinates from the supplementary data of SRAMP (37) to Ensembl database v79. Only human data from these datasets were used to evaluate our models, and only the m^6^A sites that conform to the DRACH motifs were retained. The m^6^A sites were mapped to the longest transcript when there was isoform ambiguity. Then the positive data in the benchmark dataset were extracted from randomly selected 80% transcripts. The data from the remaining transcripts were used to construct the independent testing dataset. According to existing works, we randomly sampled negative data in the benchmark dataset to keep the positive-negative ratio as 1:1. The ratio in the independent testing dataset is 1:10.

We constructed a third dataset for building a more robust m^6^A predictor by integrating the majority of currently available m^6^A sites detected by various epitranscriptome profiling technologies, denoted as robust m^6^A sites. In particular, we collected 20 datasets generated from 9 different m^6^A profiling approaches (Supplementary Table S1), constructed technique-specific epitranscriptomes by merging datasets generated from each technique, and selected those sites that can be detected by multiple techniques. Based on permutation analysis and the control of FDR, 1,243 m^6^A sites located on 933 genes that can be detected by at least 4 techniques were used to construct a technically robust benchmark dataset (Supplementary Table S2). The negative data was sampled from the DRACH motifs on the same transcripts carrying the positive sites. We excluded sites included in the m^6^A sites collected (i.e. those that were once identified as modifiable).

To evaluate Geo2vec on m^6^A signal detection from direct RNA sequencing, we downloaded HEK293T Nanopore RNA sequencing data from xPore (63). Since the next-generation sequencing-based m^6^A profiling technique m6ACE-seq (64) was proposed by the same laboratory on the same cell line, we constructed a fourth dataset using m6ACE-reported sites as training dataset to maximize concordance between data and labels. A total of 15,871 sites at DRACH were collected. All other DRACH motifs from sample transcripts as those sites reported by m6ACE-seq and not reported as methylated in any study were used as negative data (n = 234,006). For Nanopore sequencing data, all three HEK293T wild-type replicates were merged for use. Raw fast5 files were first basecalled using Guppy 3.1.5 and then resquiggled using Tombo. Inside Tombo, reads were aligned to the transcriptome with minimap2 (65) using Ensembl release version 104.

We collected 25 tissue-specific m^6^A datasets from 8 existing works, as shown in Supplementary Table S3. The raw sequencing data were downloaded from NCBI GEO (https://www.ncbi.nlm.nih.gov/geo/) (66) and National Genomics Data Center (https://ngdc.cncb.ac.cn/) (67). We used Trim Galore (68) to filter adaptors and low-quality nucleotides and HISAT2 (69) to align the processed reads to the reference genome UCSC hg19. Finally, exomePeak2 (70) was used to detect the m^6^A enriched regions (peaks) with the default setting. These called peaks were considered as positive data (regions with m^6^A signals). In our training, we further selected peaks with a width <400-nt and retained the peaks whose start and end are on exons. The negative data were randomly selected from non-peak regions of the same transcript of positive data. The positive-to-negative ratio was kept as 1:1. The negative regions were also cropped to match the peak width of positive data.

We collected the m^1^A epitranscriptome detected in the HEK293T cell line reported by four different technologies, as shown in Supplementary Table S4. The reported m^1^A sites were pooled together as positive data, and the negative sites were generated in the same way as previously described in the m^6^A prediction task.

### Geographic encoding of RNA transcripts

Of interest here is to faithfully encode the sub-molecular geographic information of ribonucleotide with respect to the entire transcript structure, such as 5′UTR, intron, and exon. Since RNA modification's functions are intrinsically associated with specific RNA regions, taking advantage of this layer of information should provide novel insights into epitranscriptome regulation.

**Figure 1.**
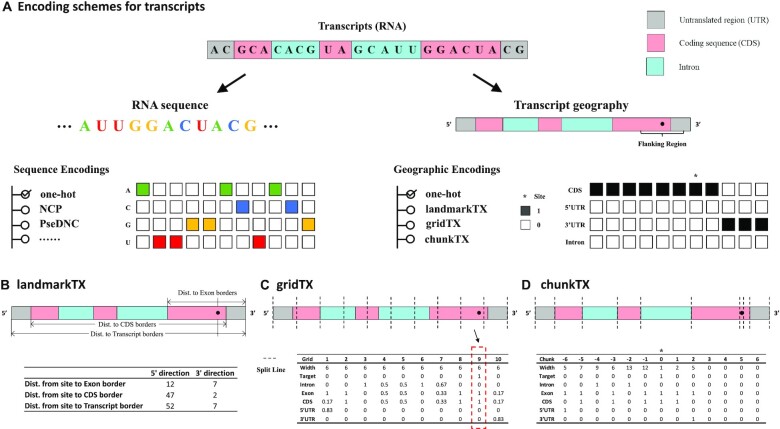
Simple graphical illustrations of the transcript descriptors. (**A**) RNA transcripts can be encoded by their sequence and geography (region types, such as 3′UTR, CDS, intron). One-hot encoding of transcript primary sequence and region type, which stacks the one-hot indicator of each nucleotide into a single feature matrix, has been widely used in machine learning applications concerning RNA. However, one-hot encoded region type features contain lots of repetitive information, shown as identical columns adjacent to each other, suggesting that the method is still inefficient. Meanwhile, trimming the sequence for a local fragment is often necessary for model selection purposes, which inevitably discard useful information. (**B**) The landmarkTX encoding uses three sets of distances to indicate the relative position of the ribonucleotide of interest on exons, CDSs, and transcripts, which not only indicates its position on the entire transcript related to key regions but also inexplicitly conveys the width of these regions. (**C**) The gridTX encoding divides a transcript into multiple fragments of equal width and returns the composition of each region type within a fragment, the fragment width, and whether a fragment contains the target ribonucleotide. (**D**) The chunkTX encoding constructs indicators at the region (with different width) level, with the target site or region in the center and zero paddings on the short side. It avoids the blurring of precise boundaries of regions and their composition. It also unambiguously retains the complete geography of the transcript and may be viewed as a condensed version of one-hot encoding after merging adjacent repetitive columns.

We assumed firstly that the relative positions of the target site (or the ribonucleotide of interest) with respect to different transcript regions of RNA are essential attributes and should be explicitly conveyed through the designed encoding scheme. To this end, we developed the first and the most straightforward encoding method that contains the above information, namely landmarkTX (Figure 1B). From local to global, three types of regions related to transcript structure are considered, including the exon, the coding sequence (CDS), and the entire transcript. The landmarkTX method presents the distance of the target site to six transcript landmarks related to three types of regions, i.e. the 5′ and 3′ boundaries of the exon, the CDS, and the entire transcript. Each distance has two directions, towards the transcript starting site (TSS) and towards the transcript ending site (TES) of RNA. The distance to the exon boundary and to the transcript boundary is always positive. When the site is located at the 5′UTR or the 3′UTR of a transcript (outside the CDS region), a negative sign is assigned to the distance to the CDS start site or CDS end site, respectively. Such a design has the following two benefits. The distances in two directions together can locate the relative position of the site on regions. Meanwhile, the model can easily learn the length of the corresponding region by adding the distances in the two directions. With only six features, landmarkTX encoding is very lightweight and very efficient.

While landmarkTX provides a most concise way to encode the sub-molecular geography of a ribonucleotide with respect to three key regions (exon, CDS, and transcript), methods that can capture the entire transcript are also of interest. Inspired by the one-hot encoded region type features (Figure 1A), we extend the indicator from single nucleotide resolution to fragment level (of the same width), named after gridTX (Figure 1C). For gridTX, the width of the fragments is a hyper-parameter and should be specified for the descriptor. The rationale behind this design is that, dividing a transcript evenly into a fixed number of fragments ensures that the information of all regions is retained in the same-shaped descriptors for all transcripts despite the difference in their length (with the width of the fragments accommodating the length difference). For each fragment, the region type composition is calculated according to the number of ribonucleotides belonging to a specific region. Five regions are considered here, including exon, intron, CDS, 5′UTR and 3′UTR. The composition of every fragment is individually evaluated. When a fragment contains the target site or a part of the target area, an indicator is added as a new layer of the descriptor. The model should learn the local context and the relative position of the target on the transcript by combining the target indicator and feature matrix. It may be worth noting that, the one-hot encoded region type feature may be considered as a special case of gridTX when the width of each fragment is set to one nucleotide (or the number of fragments is equal to the length of the transcript).

Unlike gridTX, chunkTX is constructed at the region (of different width) level and can thus avoid the blurring of the precise region boundaries and their region type composition (Figure 1D). Importantly, the length of its output depends only on the complexity (number of exons) of the transcript but not its sequence length, which makes it very efficient for describing a large trunk of ribonucleotide with the same region type. In practice, 729 regions (corresponding to a feature matrix of 729 × 6) are sufficient to accurately encode the geography of the most complex human transcript of 2 304 640-nt recorded in the Ensembl (71) transcriptome annotation database *EnsDb.Hsapiens.v79*, compared with the one-hot encoding method that requires a feature matrix of 5 × 2 million. It is important to note that chunkTX retains all the information of the entire transcript unambiguously and may be viewed as a condensed version of one-hot encoding with the adjacent repetitive features merged together. Due to varying exon numbers of transcripts, to obtain the same shaped geographic features, the use of chunkTX requires zero-padding for simple transcripts and trimming for very complex transcripts, just as the widely used one-hot encoding. Additionally, instead of using the target indicator to give the position of the target nucleotide, chunkTX aligns the target site (or area) in the middle of the feature matrix. It is worth mentioning that when the target ribonucleotide (or the entire target area) is entirely within a genomic region, the target divides the mapped region into three sub-regions, and each will be encoded independently. For instance, when a base-resolution m^6^A site is mapped within an exon, the target site itself is encoded as a region with a width of 1 and an exon indicator of 1. Additionally, the left and the right chunks are encoded as well. In general, chunkTX records the information about all regions in the transcript, including their width, composition, and relative order.

### Model design

In this work, all Geo2vec encodings, one-hot encoded region type features, and one-hot encoded sequence features were generated by our *Geo2vec* R package. The classical sequence features (NCP and PseDNC) were generated through iLearnplus (72). Both machine learning algorithms and deep learning frameworks were utilized to evaluate the transcript descriptors developed using Geo2vec. The reported results based on XGBoost model were based on Python package xgboost 1.4.2 with the default parameters. All deep learning models were constructed under Tensorflow 2.3.2.

**Figure 2.**
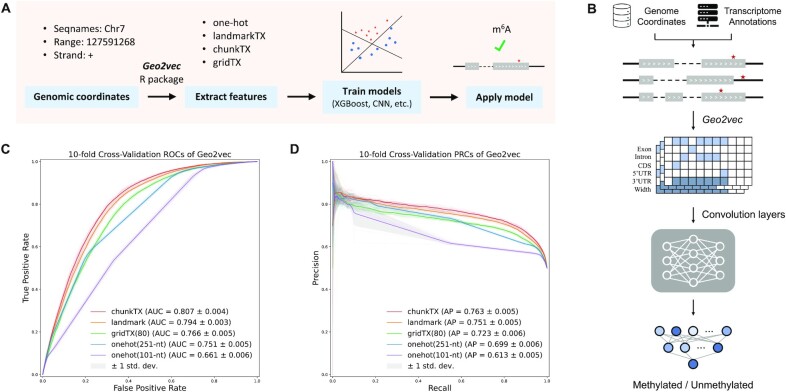
Geographic information-based m^6^A site prediction. (**A**) An overview of the machine learning pipeline. Geographic information of RNA transcripts was extracted using the *Geo2vec* R package. Models like XGBoost and CNN were trained on the generated geographic information and applied to the testing datasets. (**B**) The neural network framework used in this study. The transcript annotation used was extracted from the R/Bioconductor package *EnsDb.Hsapiens.v79*. (**C**) Receiver operating characteristic curves (ROCs) for models based on different geographic encoding methods. AUC: area under ROC curves. (**D**) Precision-recall curves (PRCs) for different transcript descriptors. AP: average precision. Ten-fold cross-validation results are given in the form of mean and standard deviation. ChunkTX is reported as the best geographic encoder of transcripts for capturing the complete transcript landscape and achieving the best prediction performance (mean AUC is 5% higher than one-hot encoding with 251-nt) and with a much lower feature dimension.

The networks used in DeepPromise (25) and DeepRiPe (61) were reproduced to show the model performances that are based on sequence features only and a combination of sequence and region type features. Both sequence and region type features were represented using one-hot encoding (e.g. A – [1, 0, 0, 0], C – [0, 1, 0, 0], G – [0, 0, 1, 0], U – [0, 0, 0, 1]). The network framework used for Geo2vec transcript descriptors (gridTX and chunkTX) is shown in Figure 2B. Two convolutional layers were used to extract features, with one max-pooling layer and one dropout layer in the middle. The number of filters was 64 and 32, and the size of kernels was 5 and 3. When combining sequence features and geographic encodings (GepSe), we adopted the multi-model framework used in DeepRiPe, but replaced the sequence module with the network used in DeepPromise and replaced the region module with the network shown in Figure 2B. A simplified graphical illustration of the network architecture and a network parameters table can be found in Supplementary Figure S1.

As for tissue-specific MeRIP-seq based m^6^A data, only coarse-grained labels are available, which means that we only know whether a peak (genome bin) contains m^6^A sites or not, but we do not know which adenosine is modifiable. We previously developed WeakRM (45), a weakly supervised learning framework that takes genome bin data of various widths as input and learns context-specific RNA methylation patterns. In our tissue-specific m^6^A prediction problem, the instance length was set to 50, and the instance stride was set to 10. All network parameters and training settings were consistent with those used in the WeakRM paper. When using the transcript descriptor (chunkTX) to assist model learning, we modified the multi-model used in base-resolution prediction by replacing the sequence module with the feature extraction network in WeakRM.

**Figure 3.**
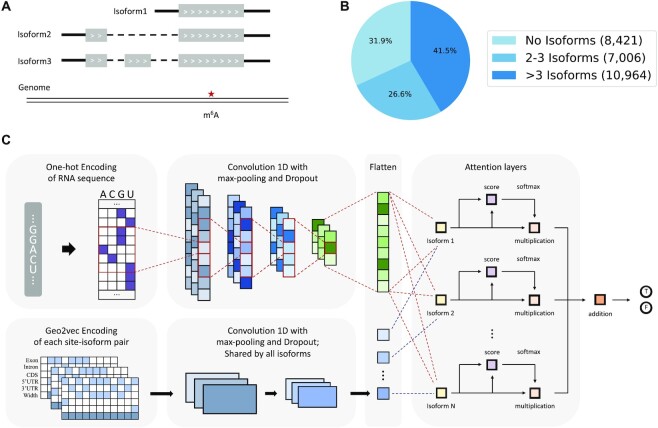
Multiple instance learning framework models isoform ambiguity. (**A**) Existing approaches typically report the genome-based coordinates of RNA modification; however, as there exist multiple isoform transcripts, it is often unclear which specific isoform transcript carries the modification. For the above-given example, although we know the genome-based coordinates of the m6A site, it is unclear which isoform transcript actually carries it. This isoform ambiguity problem exists in all existing mRNA modification databases. (**B**) A pie chart showing the number of mapped isoforms of the m^6^A site in the sramp17 benchmark dataset. No isoforms: the site overlaps with only one transcript; 2–3 Isoforms: the site overlaps with two or three transcripts; >3 Isoforms: the site overlaps with more than three transcripts. (**C**) The proposed attention-based multiple instance learning framework (i-GepSe) that makes full use of all mapped isoforms to predict m^6^A sites.

The above framework can only handle one transcript isoform at a time, thus lacking the ability to deal with isoform ambiguities. Inspired by multiple instance learning, a kind of weakly supervised learning, we treat each isoform as an instance and use an attention mechanism to merge features learned from all isoforms to obtain the final output. As shown in Figure 3C, each isoform is fed to a transcript module for feature extraction. The weights in these transcript modules are shared. The input sequence is either sent to the sequence module when base-resolution m^6^A data is available or divided into instances and learned by the MIL framework when working on genome bin data. The sequence feature is then broadcast to the same number of isoforms and concatenated with each isoform feature. The concatenated features are then fed to an attention layer. Specifically, two fully connected layers with tanh and sigmoid activation functions respectively are used to obtain a query vector for each sequence-isoform complex. Then another fully connected layer is applied to measure the similarity between the context vector (key) and the query vector and return an attention weight for each sequence-isoform complex. The learned weights are used to merge all the hidden features and generate the final output.

The read features based network for direct RNA sequencing modeling were adapted from DeepSignal (73) and DeepSignal-plant (74). One-hot encoding of nucleotide, normalized mean, standard deviation, median, median absolute deviation (MAD), and dwelling time of the signal for each nucleotide in $k$-mers centering on the site of interest ($k$ = 13 by default) were extracted as sequence features. Sampled $m$ signal values with zero paddings for each nucleotide in $k$-mers were also used as signal features. For each read, a geographic feature matrix was generated and truncated according to the start and end of the read. We followed m6Anet (Hendra *et al.* 2021) to sample 20 reads for each site and used Noisyor function to aggregate read level probability scores to site-level scores. However, for those labeled sites that can only be covered by <20 reads, we did not set a minimum read threshold of 20 but used zero padding so that our model could also capture low-expression sites. A simplified graphical illustration of network architecture can be seen in Figure 6.

The training of the base-resolution m^6^A predictor, with random or longest isoform, was conducted using a mini-batch size of 128 for 20 epochs. When multiple isoforms were considered, or the genome bin data was used, the number of epochs was reduced to 1 since the number of inputs was inconsistent in the dataset. During training, the Adam optimizer was used to minimize the binary cross-entropy loss. All training and evaluation were performed on 1 NVIDIA RTX 2080Ti.

### Evaluation scores

We evaluated the tested models using cross-validation. We used 10-fold for the base-resolution m^6^A data and 5-fold for the tissue-specific m^6^A peak data, because some tissues have relatively less positive data than the site at the single-nucleotide level. The predictions for independent datasets were obtained by averaging the outputs of all 10 cross-validation models. The model performance was assessed using the receiver operating characteristic (ROC) curves and precision-recall (PR) curves, as well as the area under ROC curves (AUC) and average precision (AP) that is defined by the weighted mean of precision scores under each threshold (the increase in recall from the previous threshold is used as weight). Average precision is more appropriate for the sramp17 independent dataset where the positive-to-negative ratio is 1:10, because it does not consider the true negatives, preventing model performance bias due to the dominance of negative data. In cross-validation, the mean scores and standard deviations of the evaluation metrics across folds were reported.

### Model interpretation

Shapley additive explanations (SHAP) (75) is used to assess the contribution of each feature to the model output. SHAP assigns each feature an importance score using the classical Shapley values from game theory and its extensions, and provides tools to combine the local interpretation of each prediction to understand the global model structure. We used SHAP to explain tree-based models on landmarkTX and chunkTX. First, a SHAP beeswarm plot was used to show the influence of the most important features on the model output. The values of these features are represented by colors, with red indicating high values, blue indicating low values, and purple indicating median values. The value points of each feature are located along the x-axis, showing the distribution of their impact on model output. A straight line indicating the zero influence on the model is also drawn. A positive impact means that the model prefers these feature values in the prediction of m^6^A. When the number of features is greater than 10, only the Top 9 important features are shown in the figure, as well as a summary of the remaining features. Although the beeswarm plot shows the impact of each feature sorted by feature importance, the total contribution of a feature and the difference in contribution between features are not completely clear. To this end, a SHAP bar plot was also provided. It shows the global contribution of the feature in terms of the average absolute SHAP value. The higher the value on the bar, the greater the influence of the feature on the model. We also evaluated the use of Deep SHAP in interpreting deep learning models (as shown in Supplementary Figure S2). The training dataset is used as a background, and a local interpretation of the predicted true positives is performed.

## RESULTS

### m^6^A site prediction based on geographic information alone

Given that RNA modifications are associated with specific sub-regions on RNA in their formation and functions, it is reasonable to assume that the geographic information encoded by our approaches can contribute to RNA modification prediction. To this end, we first evaluated the predictive power of geographic information alone in the m^6^A site prediction task using the sramp17 benchmark dataset (37).

We considered here the three newly proposed geographic encoding schemes (landmarkTX, gridTX and chunkTX) and also the widely used one-hot encoded region type features. Specifically, the mature RNA model was selected, which concerns only the exonic m^6^A and non-m^6^A sites on the mature mRNAs to prevent the bias introduced in polyA selection of RNA-seq library preparation. When a site can be mapped to multiple isoform transcripts of the same gene, the primary transcript (longest transcript) was selected for extracting geographic information using the newly developed *Geo2vec* R package based on Ensembl transcriptome annotation R/Bioconductor package *EnsDb.Hsapiens.v79*. As in the previous work (27–35), we sampled the same number of negative sites as the positive sites from the same m^6^A-carrying transcripts to construct a 1:1 positive-negative ratio dataset. In addition, 10-fold cross-validation was applied to make full use of the entire data for performance evaluation.

Since landmarkTX has only 6 features, instead of using a deep learning framework, we considered the machine learning model XGBoost. For the other high-dimensional encoding schemes, we constructed neural network models similar to DeepRiPe (61), except that the sequence input module was disabled to explore the predictive power of geographic information alone. As shown in Figure 2B, we use a two-layer convolutional neural network to extract hidden features for gridTX, chunkTX, or one-hot encoded geographic feature matrices. Receiver operating characteristic (ROC) curves and precision-recall (PR) curves were used to show the performance under different thresholds. The area under the ROC curve (AUC) and average precision (AP) are used to provide quantitative results. Since the experiment was conducted under the cross-validation framework, we used the average value under each threshold to draw the curve and used the gray area to show the standard deviation interval. AUC and AP were also given in the form of mean and standard deviation.

We first evaluated the impact of the number of slices for gridTX encoding. As shown in Supplementary Table S5, splitting the transcript into more fragments of equal width is generally helpful for the neural network to learn from gridTX. The model achieves a relatively marginal improvement after the number of fragments exceeds 80, so we choose the number of grids as 80 in the following performance evaluation. Similarly, we found that using the third quantile of the region numbers of all genes (35) as the chunk number of chunkTX encoding is appropriate for the test task (see Supplementary Table S6). The transcripts with fewer than 35 regions were padded with zeros on both sides, and the transcripts with more than 35 regions were trimmed. The region width in both gridTX and chunkTX is log-transformed. For one-hot encoding, two lengths were considered, 101-nt as used in iONMF (59) and iDeep (60), and 251-nt as used in DeepRiPe (61). Even the shorter version (101-nt) is of 505 dimensions (101 × 5), which is around twice the size of chunkTX features (with 35 chunks) and is roughly the same as gridTX (with 80 fragments), all of which are much heavier than landmarkTX with only six features.

As shown in Figure 2C and D, all three newly proposed geographic encoding methods outperformed the existing one-hot encoding method. As a baseline, the performance of one-hot encoded region type features differs greatly between two input lengths. It performs relatively poorly when only a 101-nt flanking window is available but produces better results after increasing to 251-nt. This is in line with the way that the region type features learning boundaries like CDS and 3′UTR junctions, i.e. it needs fragments long enough to cross the boundaries of the related regions. The gridTX (80 fragments) uses about half the number of features but obtained better prediction results than one-hot encoding (251-nt) with mean AUC 0.766 vs. 0.751 and mean AP 0.723 vs. 0.699. However, compared with landmarkTX and chunkTX, there is still a performance gap, suggesting that, although gridTX preserved the entire transcript landscape, bin-based information compression introduced blur and noise that undermined its learning capacity. The landmarkTX encoding with only six features achieved the second-best performance after only chunkTX. It allows the model to directly see the distance from the target site to six landmarks on the transcript, including the stop codon and the width of the exon where the target is located, which are two key features known to be strongly associated with m^6^A RNA methylation (20,21,56). Although landmarkTX already well captured the geographic characteristics of m^6^A, our results suggest that chunkTX, which captures the complete transcript landscape with no ambiguity, is currently the best geographic encoder of the transcript (mean AUC is 5% higher than one-hot encoding with 251-nt) and with a lower feature dimension (245 versus 1255).

Importantly, with an average AUC of 0.807, the models based on geographic information alone (without using the sequence information at all) are already comparable to the classic sequence-based m^6^A prediction methods such as MethyRNA (AUC of 0.791) (62) and SRAMP (AUC of 0.784) (37), highlighting the great potential of using geographic information for predicting sub-molecular events associated with RNA transcripts.

### Geographic information enhances sequence-based m^6^A predictors

After verifying the predictive power of using geographic information alone, we now examine whether the geographic encoding of transcripts can capture extra information related to RNA methylation that is missing from their sequence encodings, or in other words, whether it can enhance the performance of existing sequence-based m^6^A predictors.

Recent work on sequence-based methods has shown that, compared with machine learning models, deep neural networks can better use the input information and provide stronger predictive capabilities. Chen *et al.* conducted a comprehensive review of existing RNA modification predictors and proposed the DeepPromise method (25), which achieved state-of-the-art performance among existing deep learning models. We, therefore, use it as a baseline for sequence-based approaches. Meanwhile, DeepRiPe (61), which was designed originally for predicting RBP binding sites, provides multi-model deep neural networks that use both sequence information and one-hot encoded geographic information. We re-produced DeepPromise for sequence-only prediction and DeepRiPe for m^6^A site prediction tasks based on both sequence and region-type features. Since one-hot encoding was used as the sequence descriptor in DeepRiPe and achieved the best performance among all the compared encodings, we also applied it to encode the primary sequence in all following deep learning experiments. To test the capability of chunkTX in complementing sequence encoding, we also constructed a multi-model model GepSe (short for **Ge**ography **p**lus **Se**quences) that accepts both sequence and geographic encoding inputs (see Supplementary Figure S1). The sequence module is the same as that used in DeepPromise, which consists of 4 convolutional blocks. For the geographic module, we continue to use the two-layer convolutional network designed previously for transcript descriptor testing. The results of the two modules are flattened, merged, and sent to the output layer. The same sramp17 benchmark dataset (37) was still used in this test.

It can be seen from Table 1 that when only sequence features are used for learning, the average AUC score of the DeepPromise model is 0.864. After incorporating 251-nt length of one-hot encoded geographic features, the DeepRiPe model achieved an improvement of 1.9% in both AUC and AP. By using both sequence feature and the newly developed chunkTX encoding, the new multimodal neural network GepSe achieved the best performance with an average AUC increase of 3.2% and an average AP increase of 3.3% over the basis of DeepPromise. We also show that GepSe can help not only m^6^A prediction near stop condons, but also m^6^A at the 5′UTR (see Supplementary Table S7).

**Table 1.** Performance evaluation of Geo2vec aided models on base-resolution m^6^A prediction

Model	Features	Accuracy	*F*1-score	MCC	AUC	AP
DeepPromise	Seq	0.786 ± 0.004	0.772 ± 0.010	0.573 ± 0.009	0.864 ± 0.003	0.845 ± 0.004
DeepRiPe	Seq + Geo (one-hot)	0.812 ± 0.006	0.799 ± 0.004	0.626 ± 0.012	0.883 ± 0.004	0.864 ± 0.006
GepSe	Seq + Geo (chunkTX)	**0.822 ± 0.004**	**0.809 ± 0.007**	**0.645 ± 0.008**	**0.896 ± 0.002**	**0.878 ± 0.004**

* **Note**: Each model was trained on the same dataset using 10-fold cross-validation. The results are provided in the form of an average$ \pm$standard deviation. Bold font indicates the best performance among the three models. The threshold used for Accuracy, *F*1-score, and MCC is 0.5. AUC, the area under ROC curves; AP, average precision. Please refer to Supplementary Figure S1 for the architecture of the GepSe Model.

To further demonstrate the generalizability of the proposed geographic descriptor, we also evaluated the performance of these deep learning frameworks on the sramp17 independent testing dataset. Constructed by the SRAMP project, the independent testing dataset contains the m^6^A sites extracted from the transcripts not included in the previous benchmark dataset. The ratio of positive data to negative data is kept as 1:10. Under this unbalanced setting, average precision (AP) is considered a more powerful evaluation metric. As shown in Table 2, among all the three models, our new GepSe model that incorporates both geographic (chunkTX) and sequence features achieved the best performance (7.1% higher than DeepPromise and 3.4% higher than DeepRiPe). These results demonstrated the effectiveness of the newly developed geographic encoding scheme in enhancing the performance of sequence-based models, suggesting that geographic encoding of transcripts indeed captured extra information concerning RNA methylation that is missed by the widely used sequence encoding of RNA transcripts.

**Table 2.** Performance of Geo2vec aided models on sramp17 independent dataset

Model	Features	Accuracy	*F*1-score	MCC	AUC	AP
DeepPromise	Sequence only	0.777	0.398	0.380	0.879	0.434
DeepRiPe	Seq + Geo (one-hot)	0.802	0.432	0.419	0.896	0.471
GepSe	Seq + Geo (chunkTX)	**0.818**	**0.456**	**0.444**	**0.908**	**0.505**

* **Note**: Predicted scores of the 10 models obtained in the benchmark cross-validation were averaged to calculate the evaluation metrics. Bold font indicates the best performance among the three models. The threshold used for accuracy, *F*1-score, and MCC is 0.5. AUC, the area under ROC curves; AP, average precision.

### Isoform-aware m^6^A site prediction enabled by geographic encoding

It is important to note that, due to technical limitations, most existing approaches for profiling the epitranscriptome detect m^6^A sites with isoform ambiguity, i.e. although the genome-based coordinates of RNA modification sites are known, it is not clear which specific isoform transcript carries the modification when there exist multiple isoform transcripts that can align with the sites in their genome projected coordinates (Figure 3A). This is primarily because of the short read length of the Illumina sequencing method that is not long enough to differentiate different isoform transcripts. To the best of our knowledge, although recent development in direct RNA sequencing technology enabled isoform-specific profiling of the RNA modifications (76), all the RNA modification sites collected in existing bioinformatics databases, such as RMBase (77), MeTDB (78) and m6A-atlas (79), and used in benchmark data for RNA modification site prediction algorithms have isoform ambiguity. Although most of the existing prediction methods were based on epitranscriptome data with isoform ambiguity, the issue has not been explicitly addressed so far.

Next, we evaluated the influence of isoform transcripts on the m^6^A methylation prediction task. For this purpose, we first examined the isoform ambiguity level in the training data we used in previous experiments and found that 68% of the m^6^A sites may be associated with more than one isoform transcript (Figure 3B). In the previous analysis, we have used the geographic information extracted from the longest isoform, which is a common and convenient way for transcript-associated analysis (80,81). However, the RNA modification signals may be from other transcripts, and shorter transcripts are of interest as well, so the arrangement is clearly not optimal, and it should be of interest to explore the possibility of isoform-specific m^6^A site prediction. To this end, we developed a deep neural network under the multiple instance learning framework (i-GepSe) that makes full use of both the sequence and the geographic information extracted from all the mapped isoform transcripts (Figure 3C). i-GepSe uses the attention mechanism to learn a weight for each isoform and then performs a weighted average of all hidden representations to obtain the final output.

As shown in Table 3, the GepSe model based on the longest or a random transcript achieved very similar performance, suggesting the two contain a similar amount of geographic information related to the entire gene (all of its transcripts). By directly modeling all the isoform transcripts through the multiple instance learning framework, the new i-GepSe model achieved distinct improvement in both average AUC and AP on an already high baseline.

**Table 3.** Performance evaluation of chunkTX aided models using different isoforms

Model	Isoform	Accuracy	*F*1-score	MCC	AUC	AP
GepSe	Longest	0.822 ± 0.004	0.809 ± 0.007	0.645 ± 0.008	0.896 ± 0.002	0.878 ± 0.004
GepSe	Random	0.821 ± 0.003	0.809 ± 0.007	0.643 ± 0.006	0.895 ± 0.002	0.877 ± 0.004
i-GepSe	All	**0.828 ± 0.004**	**0.810 ± 0.010**	**0.657 ± 0.009**	**0.901 ± 0.003**	**0.882 ± 0.004**

*Note: Each model was trained using 10-fold cross-validation. Longest, the longest mapped isoform was selected to generate geographic features; Random, one random mapped isoform was selected; All, all mapped isoforms were considered in the model. The results are provided in the form of an average$ \pm$standard. Bold font indicates the best performance among the three models. The threshold used for Accuracy, *F*1-score, and MCC is 0.5. AUC, the area under ROC curves; AP, average precision.

Next, we examined the impact of isoform ambiguity when using i-GepSe isoform-aware prediction of m^6^A sites. To this end, we divided all the m^6^A sites into three groups according to their isoform ambiguity level (i.e. mapped to 1 transcript, mapped to 2–3 transcripts, and mapped to more than 3 transcripts), and then examined the performance improvement of i-GepSe due to isoform-aware geographic information compared with the original sequence-based DeepPromise model. As shown in Table 4, compared to m^6^A sites with no isoform ambiguity (improvement of 2.7% in AUC), geographic information brought more improvements when predicting m^6^A sites with a higher level of ambiguity (improvement of 4.6% in AUC for m^6^A sites that can be aligned to more than 3 isoform transcripts). Together, these results suggest that the new isoform-aware modeling of m^6^A sites is effective in improving prediction accuracy, and there should exist isoform level differences in RNA methylation patterns that make isoform-specific m^6^A site prediction desirable.

**Table 4.** Performance improvement from sequence only model to isoform-aware model

Num. Isoform	Accuracy	*F*1-score	MCC	AUC	AP
No isoforms	+0.031	+0.033	+0.064	+0.027	+0.028
2 or 3	+0.042	+0.041	+0.085	+0.038	+0.034
Above 3	+0.056	+0.053	+0.112	+0.046	+0.046

*Note: Values for all evaluation metrics can be found in Supplementary Table S8. The results were obtained by integrating all testing datasets from each fold. That is, the entire sramp17 benchmark was used to evaluate performance improvements. The threshold used for Accuracy, *F*1-score, and MCC is 0.5. AUC, the area under ROC curves; AP, average precision.

Note that the attention mechanism used in our i-GepSe model indicates which features the model is more concerned about. Since we implemented the attention layer on isoform-level features, the learned weights should directly indicate the contribution of each isoform transcript in the m^6^A prediction task or which specific isoform transcript is more likely to carry the predicted m^6^A site. In another word, the i-GepSe model is already capable of performing isoform-specific m^6^A site prediction.

### m^6^A site prediction with minimal technical bias

*N* ^6^-methyladenosine (m^6^A) can be profiled with several different high throughput sequencing approaches including, most notably, m^6^A-seq (or MeRIP-seq) and miCLIP (20,21,82,83). It has been reported that these approaches captured very similar sequence motifs. However, a substantial discrepancy has been observed previously between different epitranscriptome profiling approaches. We collected 20 datasets generated from nine different m^6^A profiling methods and constructed the technique-specific epitranscriptomes by merging datasets generated from each technique (Supplementary Table S1). A pair-wise comparison revealed that, on average, only 14.64% of the detected m^6^A sites were shared between two arbitrary methods (Supplementary Table S9). The consistency score increased slightly to 17.69% when we restricted the analysis to house-keeping genes only (84) to minimize the impact of condition-specific gene expression (Table 5), even though this analysis still cannot rule out the impact of condition-specific regulation at the epitranscriptome layer.

**Table 5.** Comparing epitranscrptomes reported from nine techniques on housekeeping genes

	m^6^A-seq	PA-m^6^A-seq	miCLIP	m^6^A-CLIP-seq	m^6^A-REF-seq	MAZTER-seq	DART-seq	m^6^ACE-seq	m^6^A-Label-seq
m^6^A-seq	2980	325	1743	1474	105	2	14	69	98
PA-m^6^A-seq	10.91%	6828	2181	1522	202	88	160	123	134
miCLIP	58.49%	31.94%	22499	9005	948	265	657	466	625
m^6^A-CLIP-seq	49.46%	22.29%	57.54%	15649	742	142	456	411	581
m^6^A-REF-seq	3.52%	5.41%	25.37%	19.86%	3736	47	103	16	67
MAZTER-seq	0.07%	1.67%	5.03%	2.70%	1.26%	5265	141	0	8
DART-seq	0.47%	4.31%	17.69%	12.28%	2.77%	3.80%	3714	10	18
m^6^ACE-seq	10.78%	19.22%	72.81%	64.22%	2.50%	0.00%	1.56%	640	37
m^6^A-Label-seq	7.88%	10.77%	50.24%	46.70%	5.39%	0.64%	1.45%	5.78%	1244

* **Note**: The diagonal elements show the total number of m^6^A sites on housekeeping genes detected by a specific technique. The elements in the upper right triangle show the number of sites detected by two techniques simultaneously. The elements in the lower left triangle show the consistency (%) between two different techniques. Let $A$ and $B$ represent the sets of m^6^A sites uncovered by two different techniques, respectively, and $| A |$ represents the total number of sites contained within a set $A$. The consistency score of two techniques is calculated by: ${s}_{A,B} = | {A \cap B} |/{\rm{min}}( {| A |,| B |} ).$

**Figure 4.**
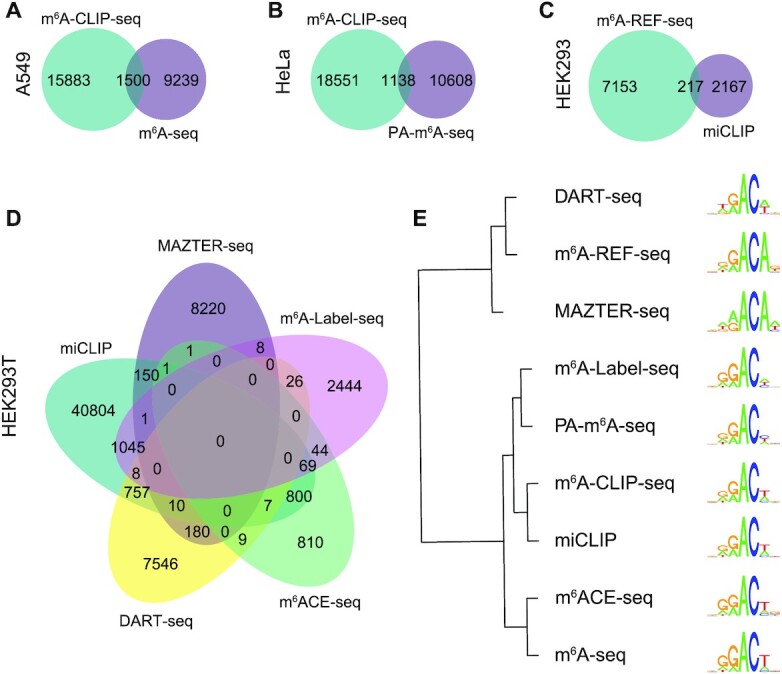
Comparison of different epitranscriptome profiling approaches in matched cell lines. (**A–D**) the Venn diagrams of the m^6^A sites uncovered by different technologies in A549, HeLa, HEK293 and HEK293T cell lines, respectively. (**E**) The sequence motifs of the m^6^A sites determined by different approaches exhibit clear clustering effects, corresponding to the functional mechanisms of the corresponding techniques, which are therefore likely to be the result of technical bias.

When we restrict the analysis to matched cell lines only, the (average) consistency scores between two arbitrary techniques are 14.00%, 6.69%, 9.10%, 12.57% in A549, HeLa, HEK293 and HEK293T cell lines, respectively (Figure 4A–D). A number of factors could contribute to the observed discrepancy among different m^6^A profiling approaches, including the functional mechanisms of the corresponding techniques, the antibody specificity caused by manufacturers and batches, differences in experimental operation, bias induced in RNA sequencing, and varying bioinformatics pipelines. For example, the antibody-based approaches m6A-seq and m6ACE-seq (64) have very similar sequence motifs, and so do the fusion-domain-based approaches MAZTER-seq (85) and DART-seq (86) (Figure 4E). These data clearly highlighted the unsuspected challenges of precise and reliable high-throughput identification of m^6^A RNA methylation sites. Naturally, it strongly encourages an integrated analysis of multiple datasets generated from orthogonal techniques to minimize the technical bias originating from a single technology.

To evaluate the statistical reproducibility between the epitranscriptomes determined by different techniques, a permutation analysis was performed on the m^6^A-forming motif DRACH in house-keeping genes. Results show that in order to obtain a false discovery rate (FDR) <0.05 with respect to technical robustness, the m^6^A sites should be observed by at least four techniques (Supplementary Table S10). This selection criterion resulted in a set of 1243 high-fidelity m^6^A sites located on 933 genes with an estimated FDR of 1.199% (Supplementary Table S2).

Based on the high-fidelity m^6^A dataset, it is now possible to construct an m^6^A site predictor with minimal technical bias. Specifically, the unmethylated adenosine used as negative data was randomly selected from the same transcripts that carry the 1243 m^6^A sites and was not identified as modifiable by any experiment. To better mimic the natural distribution of m^6^A on DRACH motifs, the positive-to-negative ratio was set to 1:10 in both training and testing datasets. Geographic encodings were generated according to the latest version of Ensembl transcriptome annotation v104. To deal with imbalanced training data in deep learning models, we up-sampled the positive sites in the training data by 10 times as in previous work (41). The test data remains unbalanced, so the most informative evaluation metric is the average precision (AP). Since only 1243 positive sites were used, we applied 5-fold cross-validation instead of 10-fold. To further demonstrate the predictive power of Geo2vec encodings, we also added the classic RNA sequence features, nucleotide chemical property (NCP) (32,36), and pseudo nucleotide composition (PseDNC) (29,30,33) used in the existing machine learning models for comparison.

As shown in Table 6, testing using an unbalanced data set, the average precision (AP) of different features can vary greatly from 0.38 to 0.88. Consistent with previous results, all three newly proposed geographic encodings outperformed the classical machine learning model based on sequence features (CNN with PseDNC and NCP encoding) and the one-hot geographic encoding. After integrating chunkTX into the sequence-based model, a substantial improvement has been achieved (AP from 0.698 to 0.881). In particular, by modeling all the mapped isoform transcripts, the average accuracy of the i-GepSe model increased further by 1.7%. Together, our results again suggested the added value of geographic information to existing sequence-based methods and the advantage of being aware of isoform ambiguity of m^6^A sites when performing the analysis. We then applied the model to predict other experimentally reported m^6^A sites and found that the greater the number of samples supporting a site, the more likely our model predicted it as methylated (Supplementary Figure S3).

**Table 6.** Performance of m^6^A site predictors with minimal technical bias

	Feature type		Performance		
Model	Sequence	Geographic	MCC	AUC	AP
XGBoost	PseDNC + NCP (51-nt)	-	0.404 ± 0.020	0.850 ± 0.012	0.422 ± 0.027
CNN	-	One-hot	0.398 ± 0.018	0.833 ± 0.009	0.376 ± 0.028
CNN	-	gridTX	0.399 ± 0.020	0.860 ± 0.006	0.435 ± 0.019
XGBoost	-	landmarkTX	0.479 ± 0.022	0.896 ± 0.007	0.491 ± 0.025
CNN	-	chunkTX (Longest)	0.473 ± 0.014	0.908 ± 0.007	0.512 ± 0.020
DeepPromise	One-hot	-	0.601 ± 0.018	0.939 ± 0.004	0.698 ± 0.031
DeepRiPe	One-hot	One-hot	0.688 ± 0.019	0.963 ± 0.007	0.806 ± 0.019
GepSe	One-hot	chunkTX (Longest)	0.766 ± 0.007	0.978 ± 0.004	0.864 ± 0.016
i-GepSe	One-hot	chunkTX (All transcripts)	**0.772 ± 0.025**	**0.981 ± 0.003**	**0.881 ± 0.013**

* **Note**: Each model was trained using 5-fold cross-validation. The results are provided in the form of an average$ \pm$standard. Bold font indicates the best performance among the models. AUC, the area under ROC curves; AP, average precision.

### Geographic information enhances tissue-specific m^6^A methylation prediction

Most of the existing approaches for m^6^A prediction ignore the context dependency of the epitranscriptome, such as cell line, tissue, and treatment. Often, the m^6^A sites detected from different biological conditions are merged together for a more complete and reliable epitranscriptome, based on which a machine learning model is constructed for general m^6^A site prediction, with the biological contexts lost. Recent studies have revealed the distinct patterns of m^6^A methylome across human tissues (87–90), which calls for condition-specific m^6^A methylation prediction methods. Although there exist multiple approaches that support context-specific m^6^A site prediction (91–94), only three human tissue types (brain, liver, and heart) are currently supported. This is due to the very limited availability of epitranscriptome datasets with base-resolution, which is required by the strongly supervised learning approaches that are dominating the field. Currently, base-resolution epitranscriptome data is available only for the human brain, liver, and heart. Without base-resolution datasets, most of the existing approaches cannot function.

**Figure 5.**
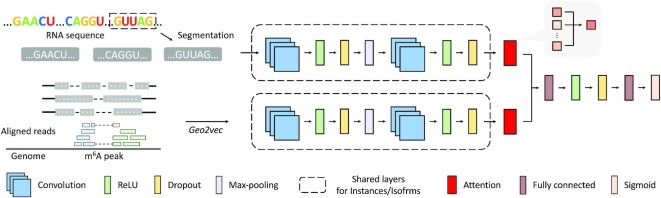
The architecture of ti-GepSe model. m^6^A peaks (low-resolution m^6^A sites) were obtained from human tissue m^6^A-seq data using exomePeak2 (70). The RNA sequence of a peak region is divided into instances of length 50 using a sliding window with a stride of 10. The instance features extracted by the convolutional layer are first merged by the attention layer to form region-level sequence features. Then, the concatenation of sequence features and geographic features of each isoform is regarded as an instance of the second multiple instance learning framework. The second attention layer is used to merge features from all isoforms and send the merged features to fully connected layers for region-level classification. It is worth noting that the ti-GepSe model retained the capability of differentiating multiple isoform transcripts.

We previously developed WeakRM (45), a weakly supervised learning framework that learns from low-resolution epitranscriptome datasets for RNA methylation patterns. As it can learn from the very widely used m^6^A-seq (MeRIP-seq) data, it is possible to use this approach to perform condition-specific m^6^A site prediction in human tissues beyond the brain, liver, and heart. We aimed to test whether geographic encoding can also enhance the performance of tissue-specific m^6^A methylation prediction in a weakly supervised learning task. For this purpose, we extracted the m^6^A methylated regions in 25 human tissues detected m^6^A-seq experiment (Supplementary Table S3) and generated matched negative control regions from the same transcripts. To enable the i-GepSe model to learn from low-resolution data, an extra layer of multiple instance learning was incorporated, as in the case of the WeakRM model, to enable tissue-specific prediction from low-resolution data. We call the new model ti-GepSe (see Figure 5). Since the number of peaks in some tissues is limited, we used 5-fold cross-validation instead of 10-fold cross-validation to ensure that each fold had enough observations to reflect the true distribution. As shown in Table 7, a substantial improvement was achieved with the new ti-GepSe model compared to the original WeakRM model with mean AUC increased from 0.813 to 0.893, and mean AP from 0.772 to 0.879. The new model achieved a score of at least 0.8 AUC in all tissues and reached 0.9 AUC in nearly half of the tissues. Interpretation of ti-GepSe also revealed tissue-specific sequence motifs for each tissue captured by our model (Supplementary Figure S4).

**Table 7.** Performance evaluation of Geo2vec aided models on tissue-specific m^6^A prediction

	AUC		AP	
Tissue type	WeakRM	ti-GepSe	WeakRM	ti-GepSe
Adrenal gland	0.788$ \pm$0.015	0.874$ \pm$0.011	0.741$ \pm$0.020	0.854$ \pm$0.012
Brainstem	0.769$ \pm$0.003	0.857$ \pm$0.006	0.716$ \pm$0.007	0.841$ \pm$0.008
Cerebellum	0.862$ \pm$0.004	0.937$ \pm$0.002	0.829$ \pm$0.007	0.935$ \pm$0.002
Cerebrum	0.850$ \pm$0.012	0.917$ \pm$0.005	0.804$ \pm$0.009	0.907$ \pm$0.006
Colon	0.778$ \pm$0.011	0.851$ \pm$0.004	0.729$ \pm$0.013	0.823$ \pm$0.006
EndoC-$\beta$H1	0.772$ \pm$0.006	0.851$ \pm$0.008	0.737$ \pm$0.010	0.831$ \pm$0.010
Endometrial	0.847$ \pm$0.008	0.927$ \pm$0.009	0.838$ \pm$0.011	0.929$ \pm$0.009
Heart	0.866$ \pm$0.008	0.929$ \pm$0.004	0.819$ \pm$0.010	0.918$ \pm$0.007
HSCs	0.736$ \pm$0.014	0.841$ \pm$0.008	0.712$ \pm$0.010	0.819$ \pm$0.013
Hypothalamus	0.781$ \pm$0.009	0.858$ \pm$0.005	0.738$ \pm$0.013	0.835$ \pm$0.006
Islet	0.856$ \pm$0.009	0.930$ \pm$0.006	0.808$ \pm$0.015	0.926$ \pm$0.008
Kidney	0.597$ \pm$0.055	0.809$ \pm$0.010	0.565$ \pm$0.062	0.798$ \pm$0.016
Liver	0.771$ \pm$0.004	0.858$ \pm$0.004	0.722$ \pm$0.007	0.832$ \pm$0.008
Lung	0.824$ \pm$0.004	0.882$ \pm$0.004	0.786$ \pm$0.007	0.862$ \pm$0.008
B-lymphocyte	0.857$ \pm$0.005	0.906$ \pm$0.005	0.812$ \pm$0.005	0.892$ \pm$0.007
Muscle	0.875$ \pm$0.010	0.933$ \pm$0.008	0.834$ \pm$0.018	0.923$ \pm$0.010
Ovary	0.890$ \pm$0.003	0.958$ \pm$0.002	0.864$ \pm$0.006	0.957$ \pm$0.002
Prostate	0.787$ \pm$0.004	0.862$ \pm$0.005	0.735$ \pm$0.009	0.840$ \pm$0.007
Rectum	0.823$ \pm$0.010	0.889$ \pm$0.008	0.783$ \pm$0.022	0.873$ \pm$0.011
RWPE-1	0.762$ \pm$0.006	0.867$ \pm$0.005	0.729$ \pm$0.011	0.864$ \pm$0.006
Skin	0.863$ \pm$0.009	0.931$ \pm$0.006	0.816$ \pm$0.015	0.920$ \pm$0.007
Stomach	0.863$ \pm$0.008	0.926$ \pm$0.006	0.822$ \pm$0.004	0.915$ \pm$0.007
Testis	0.788$ \pm$0.003	0.864$ \pm$0.005	0.744$ \pm$0.006	0.836$ \pm$0.009
Thyroid gland	0.856$ \pm$0.008	0.918$ \pm$0.002	0.796$ \pm$0.014	0.905$ \pm$0.002
Urinary bladder	0.869$ \pm$0.009	0.940$ \pm$0.003	0.826$ \pm$0.012	0.935$ \pm$0.003
Mean	0.813	**0.893**	0.772	**0.879**

*Note: Each model on each tissue data was trained using 5-fold cross-validation. The results are provided in the form of an average$ \pm$standard deviation. Bold font indicates the best performance among the three models. AUC, the area under ROC curves; AP, average precision. Gec2vec used ChunkTX encoding.

### Geographic information enhances m^6^A signal detection from direct RNA sequencing data

Oxford Nanopore direct RNA sequencing technology (ONT) provides a new solution for the detection of RNA modifications with simplified experimental procedures (95–97). ONT can capture shifts in current intensity caused by chemical modifications and thus enable supervised learning of the signal difference between modified and unmodified ribonucleotides. Existing approaches can be divided into comparative methods and supervised machine learning methods. Comparative methods, such as ELIGOS (98) and xPore (63), rely on samples with few or no m^6^A modifications. However, control samples are not always available. Supervised machine learning methods, such as EpiNano (99) and nanom6A (100), rely on labeled data from synthetic modified RNA or high throughput sequencing experiments. Since these labels are only available at the site level, most methods pool the reads down to the site level and build site-level prediction models. Given this limitation, m6Anet (Hendra *et al.*, 2021) was recently proposed to obtain both read-level and site-level probability scores using a multiple instance learning framework. We here follow the idea of m6Anet and extend geographic encoding to read-level m^6^A detection.

**Figure 6.**
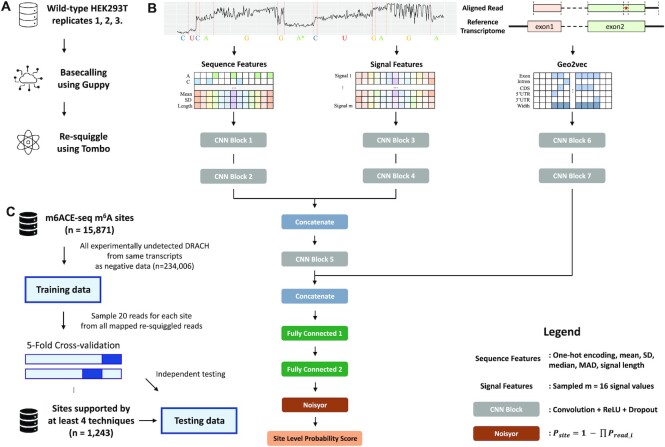
Pipeline and network architecture for Geo2vec aided m^6^A detection from direct RNA sequencing. (**A**) Data processing pipeline for Oxford Nanopore direct RNA sequencing. The raw reads were first basecalled using Guppy and then resquiggled by Tombo. (**B**) The network architecture for m^6^A detection using read features (sequence features and signal features) and geographic information with Geo2vec. (**C**) The benchmark dataset and independent testing dataset used in this study.

RNA modification stoichiometry prediction can be heavily affected by the signal-to-sequence alignment (resquiggling). As one of the two most common software, Nanopolish failed to resquiggle the reads evenly along with the same transcript and may introduce unmodified-modified proportion bias to subsequent prediction model construction (96). Tombo, which benefits from global resquiggling, can overcome these limitations and produce an increased and uniform proportion of resquiggled reads. Therefore, we chose Tombo in our data processing. For the feature representation of electronic signals, in addition to normalized mean, standard deviation, and dwelling time of the raw signal used in m6Anet, we adopted the sequence feature and signal features defined in DeepSignal (73) and DeepSignal-plant (74), which were also constructed based on Tombo resquiggled reads. Specifically, for sequence features, we constructed $k$-length features for $k$-mers centered on the target site (default $k$ = 13), including one-hot encoding of the nucleotide, normalized mean, standard deviation, median, and median absolute deviation (MAD) of each nucleotide. For signal features, we sampled $m$-length normalized signal values for each nucleotide in $k$-mers, padded it with zeros for those whose dwelling time is shorter than 16. Each feature passes through its own convolution blocks and is then concatenated for further processing (Figure 6B).

In m6Anet, only sites with at least 20 reads were used in model construction. In our model, in order to better model weak m^6^A signals on lowly expressed transcripts, we eliminated the minimum threshold of 20, and instead padded input read features with zeros to form the 20-read feature of each labeled site. For those sites covered by >20 reads, we sampled 20 reads for use. It is worth noting that the sampled reads can come from different transcripts. Then, we generated the corresponding geographic encoding for the target site from the 20 reads, resulting in 20 ChunkTX encoded geographic feature matrices as the third input (Figure 6B). Specifically, even long reads may not cover the entire transcript, so we truncate the encoding based on the actual coverage of the read to obtain a read-specific geographic encoding. These geographic features will first pass through the convolution blocks and then merge into the read features. Through two consecutive fully connected layers, the integrated features will first be transformed into read-level probability scores and finally summarized into site-level probability scores through the Noisyor function. The Nanopore direct RNA sequencing data used in this study was obtained by combining three HEK293T wild-type replicates from xPore (63). We collected the HEK293T m^6^A sites (*n* = 15 871) reported in m6ACE-seq as training labels because the samples were provided by the same lab as xPore, which should allow as much consistency as possible between labels and data. All DRACH motifs from the same transcript that have not been reported as m^6^A sites in any study were selected as negative data (*n* = 234 006).

As shown in Table 8, the model with only read features can already achieve promising performance (AUC 0.950 and AP 0.741). After incorporating the geographic information obtained from the reads, the performance of the model was significantly improved (AUC 0.977 and AP 0.817), suggesting the power of geographic information extracted from long sequencing reads. The same trend was observed in independent testing with the dataset used in Table 6 as the test dataset (Overlapping sites were removed during training). To further validate our model, we applied the model to predict all reads covering the assessed sites and generated the distribution of predicted sites on transcripts and inferred methylation levels based on the percentage of predicted methylated reads. We found that predictions for experimentally detected and undetected sites had the same distribution as existing knowledge about m^6^A and were enriched around stop codons (see Supplementary Figure S5). 92% of experimentally undetected sites were predicted to have zero methylation levels, while the majority of the remaining sites had low quantification levels, which may be due to the fact that low-stoichiometric sites are easily missed by profiling techniques. About 8% of m6ACE-seq sites had an inferred methylation level of zero, which may be partially explained by differences in methylation between samples (see Supplementary Figure S6).

**Table 8.** Performance evaluation of Geo2vec aided models on two datasets

Dataset	Features	MCC	AUC	AP
Cross validation	Read features	0.539 ± 0.017	0.950 ± 0.003	0.741 ± 0.009
	Read features + chunkTX	**0.638 ± 0.019**	**0.977 ± 0.002**	**0.817 ± 0.008**
Independent testing	Read features	0.594 ± 0.011	0.947 ± 0.002	0.825 ± 0.006
	Read features + chunkTX	**0.701 ± 0.016**	**0.977 ± 0.001**	**0.881 ± 0.004**

* **Note**: Each model was trained using 5-fold cross-validation. The results are provided in the form of an average$ \pm$standard. Bold font indicates the best performance among the models in each dataset. The positive-to-negative ratios in cross-validation and independent testing are 1:14 and 1:10, respectively. Read Features, using both sequence features and signal features. AUC, the area under ROC curves; AP, average precision.

### Geo2vec deciphers the distribution of m^6^A on transcripts through model interpretation

Although obtaining accurate predictions is important, it is often as important to understand the functional mechanisms of the prediction model and the key features behind the model decisions, so being able to interpret the role of geographic features in deep neural networks is critical. For this purpose, SHAP (Shapley additive explanations) was used to obtain the order of feature importance and allow us to understand how features affect model predictions. We examined the key features for predicting m^6^A sites located on 5′UTR, CDS and 3′UTR, respectively.

**Figure 7.**
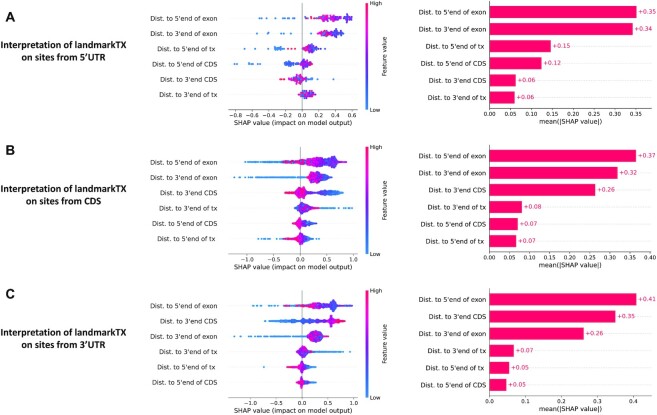
Interpretation of landmarkTX. Beeswarm plots of SHAP (Shapley additive explanations) values of every feature in landmarkTX and the corresponding bar plots of the mean absolute SHAP values (**A**–**C**). In the beeswarm plot, the features are sorted by the sum of SHAP value magnitudes. The SHAP values are used to show the distribution of the impact on model prediction of each feature (positive values indicate positive effects, and negative values indicate negative effects). The color bars show the feature value (red high, blue low). (**Note**: The sramp17 benchmark dataset was randomly divided into training and testing datasets with a ratio of 8:2. The explanation was performed on the testing data using the model trained on the training dataset. Since we used the site coordinates minus CDS start coordinates to calculate the distance from the site to the boundary of CDS (5′-end), the distance will become a negative value (for the site from the minus strand, we multiply the distance with –1 to unify the two strands) when the site is located at 5′UTR. Similarly, the distance to CDS in the 3′-end direction was calculated as the CDS end coordinates minus the site coordinates. When the site is located at 3′UTR, the distance value is negative. Therefore, in the beeswarm plot, high feature values (red dots) mean those distances are close to 0.)

The interpretation of LandmarkTX encoding was performed on XGBoost as previously implemented. As shown in Figure 7, the distances to both exon boundaries remain in the top 3 most important features in landmarkTX in all three region types and show a clear trend that the higher the distance, the more likely the model predicts the site as m^6^A modifiable. This is consistent with the fact that m^6^A modification is enriched at long exons. For the site in 5′UTR (Figure 7A), the distance to CDS in the direction of the 5′-end is clearly separable by the zero-impact axis. High distance values (distances closer to 0 since a negative sign was assigned to indicate the site is out of CDS) are clustered at the positive axis, which means that the model tends to predict the site close to the start codon as modifiable. This trend is consistent with previous results (101) that 5′UTR m^6^A modulates the start codon selection. When it comes to CDS and 3′UTR (Figure 7B and C), the distance to CDS in the 3′-end direction becomes the third or second most important feature, respectively. In the plot of CDS, we can observe that the model obviously prefers the low distance values and, the smaller the distance, the greater the impact on the model output. For 3′UTR, since a negative sign was assigned to the distance, the observed trend is as expected that the higher the distance (closer to 0), the more likely our model predicts an m^6^A site.

**Figure 8.**
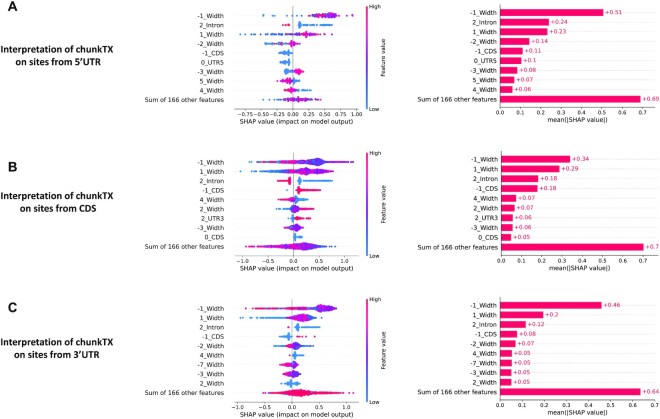
Interpretation of chunkTX. Beeswarm plots of SHAP (Shapley additive explanations) values of top 9 features (the remaining features are summed) in chunkTX and the corresponding bar plots of the mean absolute SHAP values (**A**–**C**). In the beeswarm plot, the features are sorted by the sum of SHAP value magnitudes. The SHAP values are used to show the distribution of the impact on model prediction of each feature (positive values indicate positive effects, and negative values indicate negative effects). The color bar shows the feature value (red high, blue low).

In the case of chunkTX, although the features are not clearly defined as distances, the ‘-1_width’ and ‘+1_width’ represent the width of regions next to the site (usually the two fragments of the exon that are separated by the site), equivalent to the distance to the exon boundary. These two features again contribute the most to predictions on all three region types. In particular, the width of the chunk by the 5′-end side of the site shows a dominant impact on models for the site from UTR regions (bar plots in Figure 8A and B). According to the beeswarm plot in Figure 8A, the model tends to predict that those sites on 5′UTR far from the 5′-end as modifiable, which is the same as we observed from landmarkTX. Conversely, a too large ‘-1_width’ can reduce the m^6^A prediction score for 3′UTR sites, indicating that the m^6^A sites may be enriched within a certain range of the stop codon. The region type of the second chunk on the 3′-end side of the site also contributes a lot in prediction (‘2_intron in Figure 8A–C), which provides evidence that m^6^A modification does not prefer the region adjacent to introns in the 5′-end direction. For 5′UTR, this means those 5′UTR directly connected to CDS; For CDS, this means those CDS next to the 3′UTR (can also be supported by the model's preference on positive ‘2_UTR3’); For 3′UTR, this means the terminal 3′UTR (3′UTR in the last exon). These are consistent with the findings in existing work (20,21) that the non-coding last exons are highly methylated, but not the next-to-last exons harboring the stop codon. The interpretation plot also shows that 5′UTR sites with introns on 5′-end side (high ‘-2_width’ has a positive impact on model output) and CDS sites with a wider region (most likely 3′UTR) on 3′-end side (high ‘2_width’ has a positive impact on model output) are more likely to be predicted as modifiable.

Overall, the model interpretation of geographic information provides novel insights into the m^6^A landscape on RNA transcripts, many of which are consistent with existing knowledge. Defined by distance, landmarkTX shows strong interpretability in terms of relative positions. In addition to long exons and regions near stop codons, the influence of surrounding region properties on model output was captured by chunkTX for the first time, and the bar plots showed that the remaining features contribute to the model prediction. A model explanatory analysis was also conducted for the deep learning frameworks (Supplementary Figure S2), from where similar conclusions can be achieved. It is worth noting that the above results are based on DRACH sites only, for non-DRACH m^6^A sites, please refer to Supplementary Table S11 and Supplementary Figure S7. Overall, the geographic encoding of the transcript brings additional information to models and can provide novel biological insights into the geographic relevance of m^6^A on RNA transcripts through its strong model interpretability.

## DISCUSSION

In this work, we explored different ways to encode the geography of RNA transcripts so as to capture the location of a specific ribonucleotide relevant to the entire transcript structure. Three novel, simple yet powerful transcript geographic encoding schemes were developed, including landmarkTX, gridTX, and chunkTX, which are all lighter yet capture more information than the widely applied one-hot encoded region type features.

LandmarkTX is designed as the most concise scheme that can use just six distances to represent the relative position of the target ribonucleotide on the transcript. On the other hand, gridTX and chunkTX compress the entire transcript landscape by dividing it into equal-width grids or region-level features. The overall performance of our model showed that all three newly proposed descriptors outperform the widely used one-hot encoded region type features in the m^6^A prediction task with smaller feature dimensions. In addition, they alone achieved comparable performance to classic sequence-based models.

We chose to conduct case studies on the m^6^A prediction task because it is the most studied epigenetic mark of the epitranscriptome with a strong sequence-based computational baseline and known enrichment around the stop codon (or the last exon). However, true model performance can only be reflected from reliable datasets. We carefully curated the training and validation dataset of our predictive models using the published benchmark dataset and independent testing data, as well as m^6^A sites supported by multiple epitranscriptome profiling experiments. Surprisingly, we found that out of hundreds of thousands of m^6^A sites reported so far, only 1,243 sites can be detected simultaneously by at least 4 techniques with a false discovery rate lower than 0.05. Based on this technically robust dataset, an m^6^A predictor with minimal technical bias was constructed.

Transcript isoforms have been rarely considered in computational modeling of RNA modifications. The Geo2vec encodings offer a possibility to study the influence of isoform ambiguity in identifying m^6^A sites. Models using the longest transcript or a randomly selected transcript obtained similar results, indicating that the longest transcript is not necessarily an optimal choice. An attention-based multiple instance learning framework (i-GepSe) was developed and showed that direct modeling of isoform-specific methylation helps improve model performance. Further analysis confirmed that the performance improvement brought by geographic encodings increased as the isoform ambiguity level increases, which highlighted the necessity and advantage of performing isoform-specific m^6^A methylation prediction.

The designed geographic descriptors and network frameworks were also used to promote the tissue-specific m^6^A site prediction. Since a large amount of MeRIP-seq data has been accumulated, we combined our previously developed weakly supervised learning framework WeakRM and Geo2vec transcript encodings and constructed the ti-GepSe model. Compared to the sequence-only model, the Geo2vec-aided ti-GepSe model showed a significant improvement, again demonstrating the adaptability of the descriptor to range-level data. To the best of our knowledge, this is the first work to construct high-accuracy, tissue-specific m^6^A predictors for 25 human tissues.

Recently, the Oxford Nanopore direct RNA sequencing has been used to detect modifications in RNA (63,98–100), which allows the identification of modifications at both the site and read levels for better understanding the epitranscriptome. We extended the proposed Geo2vec descriptors to the m^6^A detection from direct RNA sequencing modeling and showed significant performance improvement could be obtained by incorporating the geographic information extracted from Nanopore long reads. With the rapid development of direct RNA sequencing-based modification detection techniques, we believe that the proposed Geo2vec can provide a potential way to help measure the transcript level methylation rate and distinguish between different RNA modifications.

Explaining machine learning models is usually highly desirable. In this work, we used SHAP (Shapley additive explanations), a game-theoretic approach, to quantify and visualize each feature's contribution in identification of m^6^A methylation. It was encouraging to find that the results of our model are consistent with existing knowledge about m^6^A enrichment, including its relationship to long exons, the region around the stop codon, and the last exon (especially those containing long 3′UTR). Such results provide some evidence that the Geo2vec descriptors can allow the model to provide biological insights about the distribution of biomarkers over the RNA transcripts.

Another modification of adenosine, *N^1^*-methyladenosine (m^1^A), was also used to evaluate the effects of Geo2vec based on human m^1^A data generated from four different technologies (Supplementary Table S4). Consistent with our results on m^6^A, geographic encoding also substantially improved m^1^A prediction. While the re-trained sequence-based DeepPromise model for m^1^A achieved an AUC of 0.750, the CNN model based on ChunkTX encoding alone has obtained a very similar performance with an AUC of 0.748. By incorporating both geographic and sequence information, the GepSe model achieved an AUC of 0.842. The performance can be further improved by isoform-aware modeling of i-GepSe with an AUC of 0.855 (Supplementary Table S12). Being applicable to another RNA modification suggests the general usability of the new proposed geographic encoding schemes of transcripts, making it a powerful complement to the widely used sequence encodings in artificial intelligence applications concerning RNA transcripts by taking advantage of the widely available transcript annotations.

To best share the newly constructed Geo2vec descriptors, an R package and a web server were built to support local and online geographic feature extraction based on UCSC databases or Ensembl annotations. Our web server also supports m^6^A site prediction based on GepSe and i-GepSe with minimal technical bias. In addition, the Python code used to implement machine learning and deep learning models is also open-sourced to help explore the use of transcript descriptors.

It is worth noting that the designed transcript descriptors so far cover only basic sub-regions (exon, intron, CDS, 5′UTR, and 3′UTR) and attributes (region type and width) of RNA. Conceivably, transcript encoding schemes covering additional region types, such as alternative polyadenylation sites and 5′ terminal caps, maybe more powerful. Geo2vec also has the potential to extend to other biomolecules such as DNA, where both coding regions and noncoding regions (e.g. promoter, enhancer, silencer, etc.) can be considered. We will explore improved designs for both general and task-specific applications in our future work.

## DATA AVAILABILITY

All data used in this study is already publicly available in the GEO database and National Genomics Data Center. From the GEO database, single-base resolution m^6^A can be collected from GSE63753 and GSE71154. All accession numbers for tissue-specific m^6^A and m^1^A data can be found in Supplementary Tables S3 and S4, respectively. All the data is also available from the authors upon reasonable request.

The deep learning frameworks were implemented using Tensorflow 2.3.2, and the Python codes can be freely accessed at https://github.com/daiyun02211/Geoplus. The user-friendly Geo2vec R package developed for easy access to our novel encoding schemes (landmarkTX, gridTX and chunkTX) is publicly available at https://github.com/daiyun02211/Geo2vec. The web server for technically robust human m^6^A site prediction and extraction of all four encodings based on common annotations can be assessed from: https://www.xjtlu.edu.cn/biologicalsciences/geo2vec.

## Supplementary Material

gkac830_Supplemental_Files

## References

[1] Grosjean H. Fine-Tuning of RNA Functions by Modification and Editing. 2005; Berlin, Heidelberg Springer.

[2] Duan H.C. , Wang Y., Jia G. Dynamic and reversible RNA N(6) -methyladenosine methylation. Wiley Interdiscip. Rev. RNA. 2019; 10:e1507. 30252201 10.1002/wrna.1507

[3] Zaccara S. , Ries R.J., Jaffrey S.R. Reading, writing and erasing mRNA methylation. Nat. Rev. Mol. Cell Biol. 2019; 20:608–624. 31520073 10.1038/s41580-019-0168-5

[4] Delaunay S. , Frye M. RNA modifications regulating cell fate in cancer. Nat. Cell Biol. 2019; 21:552–559. 31048770 10.1038/s41556-019-0319-0

[5] Roundtree I.A. , Evans M.E., Pan T., He C. Dynamic RNA modifications in gene expression regulation. Cell. 2017; 169:1187–1200. 28622506 10.1016/j.cell.2017.05.045 PMC5657247

[6] Boccaletto P. , Machnicka M.A., Purta E., Piatkowski P., Baginski B., Wirecki T.K., de Crécy-Lagard V., Ross R., Limbach P.A., Kotter A. et al. MODOMICS: a database of RNA modification pathways. 2017 update. Nucleic Acids Res. 2018; 46:D303–D307. 29106616 10.1093/nar/gkx1030 PMC5753262

[7] Zhao B.S. , Roundtree I.A., He C. Post-transcriptional gene regulation by mRNA modifications. Nat. Rev. Mol. Cell Biol. 2017; 18:31–42. 27808276 10.1038/nrm.2016.132 PMC5167638

[8] Jia G. , Fu Y., He C. Reversible RNA adenosine methylation in biological regulation. Trends Genet. 2013; 29:108–115. 23218460 10.1016/j.tig.2012.11.003 PMC3558665

[9] Wang X. , Zhao B.S., Roundtree I.A., Lu Z., Han D., Ma H., Weng X., Chen K., Shi H., He C. N(6)-methyladenosine modulates messenger RNA translation efficiency. Cell. 2015; 161:1388–1399. 26046440 10.1016/j.cell.2015.05.014 PMC4825696

[10] Patil D.P. , Chen C.K., Pickering B.F., Chow A., Jackson C., Guttman M., Jaffrey S.R. m(6)A RNA methylation promotes XIST-mediated transcriptional repression. Nature. 2016; 537:369–373. 27602518 10.1038/nature19342 PMC5509218

[11] Bertero A. , Brown S., Madrigal P., Osnato A., Ortmann D., Yiangou L., Kadiwala J., Hubner N.C., de Los Mozos I.R., Sadée C. et al. The SMAD2/3 interactome reveals that TGFβ controls m(6)A mRNA methylation in pluripotency. Nature. 2018; 555:256–259. 29489750 10.1038/nature25784 PMC5951268

[12] Geula S. , Moshitch-Moshkovitz S., Dominissini D., Mansour A.A., Kol N., Salmon-Divon M., Hershkovitz V., Peer E., Mor N., Manor Y.S. et al. m6A mRNA methylation facilitates resolution of naïve pluripotency toward differentiation. Science. 2015; 347:1002–1006. 25569111 10.1126/science.1261417

[13] Fustin J.M. , Doi M., Yamaguchi Y., Hida H., Nishimura S., Yoshida M., Isagawa T., Morioka M.S., Kakeya H., Manabe I. et al. RNA-methylation-dependent RNA processing controls the speed of the circadian clock. Cell. 2013; 155:793–806. 24209618 10.1016/j.cell.2013.10.026

[14] Zhou J. , Wan J., Gao X., Zhang X., Jaffrey S.R., Qian S.B. Dynamic m(6)A mRNA methylation directs translational control of heat shock response. Nature. 2015; 526:591–594. 26458103 10.1038/nature15377 PMC4851248

[15] Niu Y. , Lin Z., Wan A., Chen H., Liang H., Sun L., Wang Y., Li X., Xiong X.F., Wei B. et al. RNA N6-methyladenosine demethylase FTO promotes breast tumor progression through inhibiting BNIP3. Mol. Cancer. 2019; 18:46. 30922314 10.1186/s12943-019-1004-4 PMC6437932

[16] Lin S. , Liu J., Jiang W., Wang P., Sun C., Wang X., Chen Y., Wang H. METTL3 promotes the proliferation and mobility of gastric cancer cells. Open Med (Wars). 2019; 14:25–31. 30886897 10.1515/med-2019-0005 PMC6419388

[17] Zhuang C. , Zhuang C., Luo X., Huang X., Yao L., Li J., Li Y., Xiong T., Ye J., Zhang F. et al. N6-methyladenosine demethylase FTO suppresses clear cell renal cell carcinoma through a novel FTO-PGC-1α signalling axis. J. Cell. Mol. Med. 2019; 23:2163–2173. 30648791 10.1111/jcmm.14128 PMC6378205

[18] Han D. , Liu J., Chen C., Dong L., Liu Y., Chang R., Huang X., Liu Y., Wang J., Dougherty U. et al. Anti-tumour immunity controlled through mRNA m(6)A methylation and YTHDF1 in dendritic cells. Nature. 2019; 566:270–274. 30728504 10.1038/s41586-019-0916-x PMC6522227

[19] Sarkar A. , Gasperi W., Begley U., Nevins S., Huber S.M., Dedon P.C., Begley T.J. Detecting the epitranscriptome. Wiley Interdiscip. Rev. RNA. 2021; 12:e1663. 33987958 10.1002/wrna.1663

[20] Meyer K.D. , Saletore Y., Zumbo P., Elemento O., Mason C.E., Jaffrey S.R. Comprehensive analysis of mRNA methylation reveals enrichment in 3′ UTRs and near stop codons. Cell. 2012; 149:1635–1646. 22608085 10.1016/j.cell.2012.05.003 PMC3383396

[21] Dominissini D. , Moshitch-Moshkovitz S., Schwartz S., Salmon-Divon M., Ungar L., Osenberg S., Cesarkas K., Jacob-Hirsch J., Amariglio N., Kupiec M. et al. Topology of the human and mouse m6A RNA methylomes revealed by m6A-seq. Nature. 2012; 485:201–206. 22575960 10.1038/nature11112

[22] Zhu X. , He J., Zhao S., Tao W., Xiong Y., Bi S. A comprehensive comparison and analysis of computational predictors for RNA N6-methyladenosine sites of saccharomyces cerevisiae. Brief. Funct. Genomics. 2019; 18:367–376. 31609411 10.1093/bfgp/elz018

[23] Lv H. , Zhang Z.M., Li S.H., Tan J.X., Chen W., Lin H. Evaluation of different computational methods on 5-methylcytosine sites identification. Brief. Bioinform. 2020; 21:982–995. 31157855 10.1093/bib/bbz048

[24] Chen X. , Sun Y.Z., Liu H., Zhang L., Li J.Q., Meng J. RNA methylation and diseases: experimental results, databases, web servers and computational models. Brief. Bioinform. 2019; 20:896–917. 29165544 10.1093/bib/bbx142

[25] Chen Z. , Zhao P., Li F., Wang Y., Smith A.I., Webb G.I., Akutsu T., Baggag A., Bensmail H., Song J. Comprehensive review and assessment of computational methods for predicting RNA post-transcriptional modification sites from RNA sequences. Brief. Bioinform. 2020; 21:1676–1696. 31714956 10.1093/bib/bbz112

[26] El Allali A. , Elhamraoui Z., Daoud R. Machine learning applications in RNA modification sites prediction. Comput. Struct. Biotechnol. J. 2021; 19:5510–5524. 34712397 10.1016/j.csbj.2021.09.025 PMC8517552

[27] Qiu W.R. , Jiang S.Y., Sun B.Q., Xiao X., Cheng X., Chou K.C. iRNA-2methyl: identify RNA 2′-O-methylation sites by incorporating sequence-coupled effects into general PseKNC and ensemble classifier. Med. Chem. 2017; 13:734–743. 28641529 10.2174/1573406413666170623082245

[28] Yang H. , Lv H., Ding H., Chen W., Lin H. iRNA-2OM: a sequence-based predictor for identifying 2′-O-methylation sites in homo sapiens. J. Comput. Biol. 2018; 25:1266–1277. 30113871 10.1089/cmb.2018.0004

[29] Chen W. , Ding H., Zhou X., Lin H., Chou K.C. iRNA(m6A)-PseDNC: identifying N(6)-methyladenosine sites using pseudo dinucleotide composition. Anal. Biochem. 2018; 561-562:59–65. 30201554 10.1016/j.ab.2018.09.002

[30] Chen W. , Feng P., Ding H., Lin H., Chou K.C. iRNA-Methyl: identifying N(6)-methyladenosine sites using pseudo nucleotide composition. Anal. Biochem. 2015; 490:26–33. 26314792 10.1016/j.ab.2015.08.021

[31] Qiu W.R. , Jiang S.Y., Xu Z.C., Xiao X., Chou K.C. iRNAm5C-PseDNC: identifying RNA 5-methylcytosine sites by incorporating physical-chemical properties into pseudo dinucleotide composition. Oncotarget. 2017; 8:41178–41188. 28476023 10.18632/oncotarget.17104 PMC5522291

[32] Chen W. , Song X., Lv H., Lin H. iRNA-m2G: identifying N(2)-methylguanosine sites based on sequence-derived information. Mol. Ther. Nucleic Acids. 2019; 18:253–258. 31581049 10.1016/j.omtn.2019.08.023 PMC6796771

[33] Chen W. , Feng P., Song X., Lv H., Lin H. iRNA-m7G: identifying N(7)-methylguanosine sites by fusing multiple features. Mol. Ther. Nucleic Acids. 2019; 18:269–274. 31581051 10.1016/j.omtn.2019.08.022 PMC6796804

[34] Tahir M. , Tayara H., Chong K.T. iRNA-PseKNC(2methyl): identify RNA 2′-O-methylation sites by convolution neural network and chou's pseudo components. J. Theor. Biol. 2019; 465:1–6. 30590059 10.1016/j.jtbi.2018.12.034

[35] Chen W. , Tang H., Ye J., Lin H., Chou K.C. iRNA-PseU: identifying RNA pseudouridine sites. Mol. Ther. Nucleic Acids. 2016; 5:e332. 28427142 10.1038/mtna.2016.37 PMC5330936

[36] Feng P. , Chen W. iRNA-m5U: a sequence based predictor for identifying 5-methyluridine modification sites in saccharomyces cerevisiae. Methods. 2021; 203:28–31. 33882361 10.1016/j.ymeth.2021.04.013

[37] Zhou Y. , Zeng P., Li Y.H., Zhang Z., Cui Q. SRAMP: prediction of mammalian N6-methyladenosine (m6A) sites based on sequence-derived features. Nucleic Acids Res. 2016; 44:e91. 26896799 10.1093/nar/gkw104 PMC4889921

[38] Chen K. , Wei Z., Zhang Q., Wu X., Rong R., Lu Z., Su J., de Magalhães J.P., Rigden D.J., Meng J. WHISTLE: a high-accuracy map of the human N6-methyladenosine (m6A) epitranscriptome predicted using a machine learning approach. Nucleic Acids Res. 2019; 47:e41. 30993345 10.1093/nar/gkz074 PMC6468314

[39] Zou Q. , Xing P., Wei L., Liu B. Gene2vec: gene subsequence embedding for prediction of mammalian n (6)-methyladenosine sites from mRNA. RNA. 2019; 25:205–218. 30425123 10.1261/rna.069112.118 PMC6348985

[40] Zhai J. , Song J., Cheng Q., Tang Y., Ma C. PEA: an integrated r toolkit for plant epitranscriptome analysis. Bioinformatics. 2018; 34:3747–3749. 29850798 10.1093/bioinformatics/bty421

[41] Xiong Y. , He X., Zhao D., Tian T., Hong L., Jiang T., Zeng J. Modeling multi-species RNA modification through multi-task curriculum learning. Nucleic Acids Res. 2021; 49:3719–3734. 33744973 10.1093/nar/gkab124 PMC8053129

[42] Körtel N. , Rücklé C., Zhou Y., Busch A., Hoch-Kraft P., Sutandy F.X.R., Haase J., Pradhan M., Musheev M., Ostareck D. et al. Deep and accurate detection of m6A RNA modifications using miCLIP2 and m6Aboost machine learning. Nucleic Acids Res. 2021; 49:e92. 34157120 10.1093/nar/gkab485 PMC8450095

[43] Song Z. , Huang D., Song B., Chen K., Song Y., Liu G., Su J., Magalhães J.P., Rigden D.J., Meng J. Attention-based multi-label neural networks for integrated prediction and interpretation of twelve widely occurring RNA modifications. Nat. Commun. 2021; 12:4011. 34188054 10.1038/s41467-021-24313-3 PMC8242015

[44] Wang C. , Ju Y., Zou Q., Lin C. DeepAc4C: a convolutional neural network model with hybrid features composed of physicochemical patterns and distributed representation information for identification of N4-acetylcytidine in mRNA. Bioinformatics. 2022; 38:52–57. 10.1093/bioinformatics/btab611 34427581

[45] Huang D. , Song B., Wei J., Su J., Coenen F., Meng J. Weakly supervised learning of RNA modifications from low-resolution epitranscriptome data. Bioinformatics. 2021; 37:i222–i230. 34252943 10.1093/bioinformatics/btab278 PMC8336446

[46] He X. , Zhang S., Zhang Y., Lei Z., Jiang T., Zeng J. Characterizing RNA pseudouridylation by convolutional neural networks. Genomics Proteomics Bioinformatics. 2021; 19:815–833. 33631424 10.1016/j.gpb.2019.11.015 PMC9170758

[47] Ao C. , Zou Q., Yu L. NmRF: identification of multispecies RNA 2'-O-methylation modification sites from RNA sequences. Brief. Bioinform. 2022; 23:bbab480. 34850821 10.1093/bib/bbab480

[48] The Gene Ontology Consortium The gene ontology resource: 20 years and still GOing strong. Nucleic Acids Res. 2019; 47:D330–D338. 30395331 10.1093/nar/gky1055 PMC6323945

[49] Senior A.W. , Evans R., Jumper J., Kirkpatrick J., Sifre L., Green T., Qin C., Žídek A., Nelson A.W.R., Bridgland A. et al. Improved protein structure prediction using potentials from deep learning. Nature. 2020; 577:706–710. 31942072 10.1038/s41586-019-1923-7

[50] McGuffin L.J. , Bryson K., Jones D.T. The PSIPRED protein structure prediction server. Bioinformatics. 2000; 16:404–405. 10869041 10.1093/bioinformatics/16.4.404

[51] Reuter J.S. , Mathews D.H. RNAstructure: software for RNA secondary structure prediction and analysis. BMC Bioinf. 2010; 11:129. 10.1186/1471-2105-11-129 PMC2984261 20230624

[52] Liu B. , Gao X., Zhang H. BioSeq-Analysis2.0: an updated platform for analyzing DNA, RNA and protein sequences at sequence level and residue level based on machine learning approaches. Nucleic Acids Res. 2019; 47:e127. 31504851 10.1093/nar/gkz740 PMC6847461

[53] Muhammod R. , Ahmed S., Md Farid D., Shatabda S., Sharma A., Dehzangi A. PyFeat: a Python-based effective feature generation tool for DNA, RNA and protein sequences. Bioinformatics. 2019; 35:3831–3833. 30850831 10.1093/bioinformatics/btz165 PMC6761934

[54] Zuo Y. , Li Y., Chen Y., Li G., Yan Z., Yang L. PseKRAAC: a flexible web server for generating pseudo K-tuple reduced amino acids composition. Bioinformatics. 2017; 33:122–124. 27565583 10.1093/bioinformatics/btw564

[55] Ao C. , Yu L., Zou Q. Prediction of bio-sequence modifications and the associations with diseases. Brief. Funct. Genomics. 2021; 20:1–18. 33313647 10.1093/bfgp/elaa023

[56] Ke S. , Alemu E.A., Mertens C., Gantman E.C., Fak J.J., Mele A., Haripal B., Zucker-Scharff I., Moore M.J., Park C.Y. et al. A majority of m6A residues are in the last exons, allowing the potential for 3′ UTR regulation. Genes Dev. 2015; 29:2037–2053. 26404942 10.1101/gad.269415.115 PMC4604345

[57] Mendel M. , Delaney K., Pandey R.R., Chen K.M., Wenda J.M., Vågbø C.B., Steiner F.A., Homolka D., Pillai R.S. Splice site m(6)A methylation prevents binding of U2AF35 to inhibit RNA splicing. Cell. 2021; 184:3125–3142. 33930289 10.1016/j.cell.2021.03.062 PMC8208822

[58] Hafner M. , Landthaler M., Burger L., Khorshid M., Hausser J., Berninger P., Rothballer A., Ascano M. Jr, Jungkamp A.C., Munschauer M. et al. Transcriptome-wide identification of RNA-binding protein and microRNA target sites by PAR-CLIP. Cell. 2010; 141:129–141. 20371350 10.1016/j.cell.2010.03.009 PMC2861495

[59] Stražar M. , Žitnik M., Zupan B., Ule J., Curk T. Orthogonal matrix factorization enables integrative analysis of multiple RNA binding proteins. Bioinformatics. 2016; 32:1527–1535. 26787667 10.1093/bioinformatics/btw003 PMC4894278

[60] Pan X. , Shen H.B. RNA-protein binding motifs mining with a new hybrid deep learning based cross-domain knowledge integration approach. BMC Bioinf. 2017; 18:136. 10.1186/s12859-017-1561-8 PMC5331642 28245811

[61] Ghanbari M. , Ohler U. Deep neural networks for interpreting RNA-binding protein target preferences. Genome Res. 2020; 30:214–226. 31992613 10.1101/gr.247494.118 PMC7050519

[62] Chen W. , Tang H., Lin H. MethyRNA: a web server for identification of N(6)-methyladenosine sites. J. Biomol. Struct. Dyn. 2017; 35:683–687. 26912125 10.1080/07391102.2016.1157761

[63] Pratanwanich P.N. , Yao F., Chen Y., Koh C.W.Q., Wan Y.K., Hendra C., Poon P., Goh Y.T., Yap P.M.L., Chooi J.Y. et al. Identification of differential RNA modifications from nanopore direct RNA sequencing with xPore. Nat. Biotechnol. 2021; 39:1394–1402. 34282325 10.1038/s41587-021-00949-w

[64] Koh C.W.Q. , Goh Y.T., Goh W.S.S. Atlas of quantitative single-base-resolution N(6)-methyl-adenine methylomes. Nat. Commun. 2019; 10:5636. 31822664 10.1038/s41467-019-13561-z PMC6904561

[65] Li H. Minimap2: pairwise alignment for nucleotide sequences. Bioinformatics. 2018; 34:3094–3100. 29750242 10.1093/bioinformatics/bty191 PMC6137996

[66] Barrett T. , Wilhite S.E., Ledoux P., Evangelista C., Kim I.F., Tomashevsky M., Marshall K.A., Phillippy K.H., Sherman P.M., Holko M. et al. NCBI GEO: archive for functional genomics data sets–update. Nucleic Acids Res. 2013; 41:D991–D995. 23193258 10.1093/nar/gks1193 PMC3531084

[67] CNCB-NGDC Members and Partners Database resources of the national genomics data center, china national center for bioinformation in 2022. Nucleic Acids Res. 2022; 50:D27–D38. 34718731 10.1093/nar/gkab951 PMC8728233

[68] Martin M. CUTADAPT removes adapter sequences from high-throughput sequencing reads. EMBnet.journal. 2011; 17:10–12.

[69] Kim D. , Paggi J.M., Park C., Bennett C., Salzberg S.L. Graph-based genome alignment and genotyping with HISAT2 and HISAT-genotype. Nat. Biotechnol. 2019; 37:907–915. 31375807 10.1038/s41587-019-0201-4 PMC7605509

[70] Meng J. , Lu Z., Liu H., Zhang L., Zhang S., Chen Y., Rao M.K., Huang Y. A protocol for RNA methylation differential analysis with merip-Seq data and exomePeak R/Bioconductor package. Methods. 2014; 69:274–281. 24979058 10.1016/j.ymeth.2014.06.008 PMC4194139

[71] Howe K.L. , Achuthan P., Allen J., Allen J., Alvarez-Jarreta J., Amode M.R., Armean I.M., Azov A.G., Bennett R., Bhai J. et al. Ensembl 2021. Nucleic Acids Res. 2021; 49:D884–D891. 33137190 10.1093/nar/gkaa942 PMC7778975

[72] Chen Z. , Zhao P., Li C., Li F., Xiang D., Chen Y.Z., Akutsu T., Daly R.J., Webb G.I., Zhao Q. et al. iLearnPlus: a comprehensive and automated machine-learning platform for nucleic acid and protein sequence analysis, prediction and visualization. Nucleic Acids Res. 2021; 49:e60. 33660783 10.1093/nar/gkab122 PMC8191785

[73] Ni P. , Huang N., Zhang Z., Wang D.P., Liang F., Miao Y., Xiao C.L., Luo F., Wang J. DeepSignal: detecting DNA methylation state from nanopore sequencing reads using deep-learning. Bioinformatics. 2019; 35:4586–4595. 30994904 10.1093/bioinformatics/btz276

[74] Ni P. , Huang N., Nie F., Zhang J., Zhang Z., Wu B., Bai L., Liu W., Xiao C.L., Luo F. et al. Genome-wide detection of cytosine methylations in plant from nanopore data using deep learning. Nat. Commun. 2021; 12:5976. 34645826 10.1038/s41467-021-26278-9 PMC8514461

[75] Lundberg S.M. , Erion G., Chen H., DeGrave A., Prutkin J.M., Nair B., Katz R., Himmelfarb J., Bansal N., Lee S.I. From local explanations to global understanding with explainable AI for trees. Nat. Mach. Intell. 2020; 2:56–67. 32607472 10.1038/s42256-019-0138-9 PMC7326367

[76] Lorenz D.A. , Sathe S., Einstein J.M., Yeo G.W. Direct RNA sequencing enables m(6)A detection in endogenous transcript isoforms at base-specific resolution. RNA. 2020; 26:19–28. 31624092 10.1261/rna.072785.119 PMC6913132

[77] Xuan J.J. , Sun W.J., Lin P.H., Zhou K.R., Liu S., Zheng L.L., Qu L.H., Yang J.H. RMBase v2.0: deciphering the map of RNA modifications from epitranscriptome sequencing data. Nucleic Acids Res. 2018; 46:D327–D334. 29040692 10.1093/nar/gkx934 PMC5753293

[78] Liu H. , Wang H., Wei Z., Zhang S., Hua G., Zhang S.W., Zhang L., Gao S.J., Meng J., Chen X. et al. MeT-DB V2.0: elucidating context-specific functions of N6-methyl-adenosine methyltranscriptome. Nucleic Acids Res. 2018; 46:D281–D287. 29126312 10.1093/nar/gkx1080 PMC5753212

[79] Tang Y. , Chen K., Song B., Ma J., Wu X., Xu Q., Wei Z., Su J., Liu G., Rong R. et al. m6A-Atlas: a comprehensive knowledgebase for unraveling the N6-methyladenosine (m6A) epitranscriptome. Nucleic Acids Res. 2021; 49:D134–D143. 32821938 10.1093/nar/gkaa692 PMC7779050

[80] Olarerin-George A.O. , Jaffrey S.R. MetaPlotR: a Perl/R pipeline for plotting metagenes of nucleotide modifications and other transcriptomic sites. Bioinformatics. 2017; 33:1563–1564. 28158328 10.1093/bioinformatics/btx002 PMC5860047

[81] Wang Y. , Chen K., Wei Z., Coenen F., Su J., Meng J. MetaTX: deciphering the distribution of mRNA-related features in the presence of isoform ambiguity, with applications in epitranscriptome analysis. Bioinformatics. 2021; 37:1285–1291. 33135046 10.1093/bioinformatics/btaa938

[82] Schwartz S. , Agarwala S.D., Mumbach M.R., Jovanovic M., Mertins P., Shishkin A., Tabach Y., Mikkelsen T.S., Satija R., Ruvkun G. et al. High-resolution mapping reveals a conserved, widespread, dynamic mRNA methylation program in yeast meiosis. Cell. 2013; 155:1409–1421. 24269006 10.1016/j.cell.2013.10.047 PMC3956118

[83] Linder B. , Grozhik A.V., Olarerin-George A.O., Meydan C., Mason C.E., Jaffrey S.R. Single-nucleotide-resolution mapping of m6A and m6Am throughout the transcriptome. Nat. Methods. 2015; 12:767–772. 26121403 10.1038/nmeth.3453 PMC4487409

[84] Eisenberg E. , Levanon E.Y. Human housekeeping genes, revisited. Trends Genet. 2013; 29:569–574. 23810203 10.1016/j.tig.2013.05.010

[85] Garcia-Campos M.A. , Edelheit S., Toth U., Safra M., Shachar R., Viukov S., Winkler R., Nir R., Lasman L., Brandis A. et al. Deciphering the “m(6)A code” via antibody-independent quantitative profiling. Cell. 2019; 178:731–747. 31257032 10.1016/j.cell.2019.06.013

[86] Meyer K.D. DART-seq: an antibody-free method for global m(6)A detection. Nat. Methods. 2019; 16:1275–1280. 31548708 10.1038/s41592-019-0570-0 PMC6884681

[87] Chatsirisupachai K. , Lesluyes T., Paraoan L., Van Loo P., de Magalhães J.P. An integrative analysis of the age-associated multi-omic landscape across cancers. Nat. Commun. 2021; 12:2345. 33879792 10.1038/s41467-021-22560-y PMC8058097

[88] Silva A.S. , Wood S.H., van Dam S., Berres S., McArdle A., de Magalhães J.P. Gathering insights on disease etiology from gene expression profiles of healthy tissues. Bioinformatics. 2011; 27:3300–3305. 21994229 10.1093/bioinformatics/btr559

[89] Pei G. , Hu R., Jia P., Zhao Z. DeepFun: a deep learning sequence-based model to decipher non-coding variant effect in a tissue- and cell type-specific manner. Nucleic Acids Res. 2021; 49:W131–W139. 34048560 10.1093/nar/gkab429 PMC8262726

[90] Tegowski M. , Flamand M.N., Meyer K.D. scDART-seq reveals distinct m(6)A signatures and mRNA methylation heterogeneity in single cells. Mol. Cell. 2022; 82:868–878. 35081365 10.1016/j.molcel.2021.12.038 PMC8857065

[91] Liu K. , Cao L., Du P., Chen W. im6A-TS-CNN: identifying the N(6)-Methyladenine site in multiple tissues by using the convolutional neural network. Mol Ther Nucleic Acids. 2020; 21:1044–1049. 32858457 10.1016/j.omtn.2020.07.034 PMC7473875

[92] Dao F.Y. , Lv H., Yang Y.H., Zulfiqar H., Gao H., Lin H. Computational identification of N6-methyladenosine sites in multiple tissues of mammals. Comput. Struct. Biotechnol. J. 2020; 18:1084–1091. 32435427 10.1016/j.csbj.2020.04.015 PMC7229270

[93] Abbas Z. , Tayara H., Zou Q., Chong K.T. TS-m6A-DL: Tissue-specific identification of N6-methyladenosine sites using a universal deep learning model. Comput. Struct. Biotechnol. J. 2021; 19:4619–4625. 34471503 10.1016/j.csbj.2021.08.014 PMC8383060

[94] Wang J. , Wang L. Deep analysis of RNA N(6)-adenosine methylation (m(6)A) patterns in human cells. NAR Genom Bioinform. 2020; 2:lqaa007. 33575554 10.1093/nargab/lqaa007 PMC7671394

[95] Qin H. , Ou L., Gao J., Chen L., Wang J.W., Hao P., Li X. DENA: training an authentic neural network model using nanopore sequencing data of arabidopsis transcripts for detection and quantification of n(6)-methyladenosine on RNA. Genome Biol. 2022; 23:25. 35039061 10.1186/s13059-021-02598-3 PMC8762864

[96] Begik O. , Lucas M.C., Pryszcz L.P., Ramirez J.M., Medina R., Milenkovic I., Cruciani S., Liu H., Vieira H.G.S., Sas-Chen A. et al. Quantitative profiling of pseudouridylation dynamics in native RNAs with nanopore sequencing. Nat. Biotechnol. 2021; 39:1278–1291. 33986546 10.1038/s41587-021-00915-6

[97] Leger A. , Amaral P.P., Pandolfini L., Capitanchik C., Capraro F., Miano V., Migliori V., Toolan-Kerr P., Sideri T., Enright A.J. et al. RNA modifications detection by comparative nanopore direct RNA sequencing. Nat. Commun. 2021; 12:7198. 34893601 10.1038/s41467-021-27393-3 PMC8664944

[98] Jenjaroenpun P. , Wongsurawat T., Wadley T.D., Wassenaar T.M., Liu J., Dai Q., Wanchai V., Akel N.S., Jamshidi-Parsian A., Franco A.T. et al. Decoding the epitranscriptional landscape from native RNA sequences. Nucleic Acids Res. 2021; 49:e7. 32710622 10.1093/nar/gkaa620 PMC7826254

[99] Liu H. , Begik O., Lucas M.C., Ramirez J.M., Mason C.E., Wiener D., Schwartz S., Mattick J.S., Smith M.A., Novoa E.M. Accurate detection of m(6)A RNA modifications in native RNA sequences. Nat. Commun. 2019; 10:4079. 31501426 10.1038/s41467-019-11713-9 PMC6734003

[100] Gao Y. , Liu X., Wu B., Wang H., Xi F., Kohnen M.V., Reddy A.S.N., Gu L. Quantitative profiling of N(6)-methyladenosine at single-base resolution in stem-differentiating xylem of populus trichocarpa using nanopore direct RNA sequencing. Genome Biol. 2021; 22:22. 33413586 10.1186/s13059-020-02241-7 PMC7791831

[101] Zhou J. , Wan J., Shu X.E., Mao Y., Liu X.M., Yuan X., Zhang X., Hess M.E., Brüning J.C., Qian S.B. N(6)-Methyladenosine guides mRNA alternative translation during integrated stress response. Mol. Cell. 2018; 69:636–647. 29429926 10.1016/j.molcel.2018.01.019 PMC5816726

