# Supplementary material for: Geographic encoding of transcripts enabled high-accuracy and isoform-aware deep learning of RNA methylation
Source: Nucleic Acids Res. 2022 Sep 26;50(18):10290–310. doi: 10.1093/nar/gkac830 (PMC9561283; doi:10.1093/nar/gkac830)
Supplement: gkac830_Supplemental_Files [file gkac830_supplemental_files.zip › Supplementary_material.pdf]

Geographic encoding of transcripts enabled high-accuracy and isoform-aware deep learning of RNA methylation

Daiyun Huang, Kunqi Chen, Bowen Song, Zhen Wei, Jionglong Su, Frans Coenen, João Pedro de Magalhães, Daniel J. Rigden and Jia Meng

Supplementary Table S1 m^{6}A epitranscriptomes obtained from different techniques

	Technique	Based on	Cell Line	PolyA	GEO	Ref
1	m6A-seq (25bp)	antibody	A549	Yes	GSE54365	(1)
2	PA-m6A-seq		HeLa	Yes	GSE54921	(2)
3	m6A-CLIP-seq		CD8T	Yes	GSE71154	(3)
4			A549	Yes	GSE71154	(3)
5			HeLa	No	GSE86336	(4)
6	miCLIP		HEK293	Yes	GSE63753	(5)
7			HEK293T	Yes	GSE122948	(6)
8			HCT116	Yes	GSE128699	(7)
9			HepG2	Yes	GSE73405	(8)
10			MOLM13	Yes	GSE98623	(9)
11			HepG2	Yes	GSE121942	(10)
12	m6ACE-seq		HEK293T	Yes	GSE124509	(11)
13	m6A-REF-seq	enzyme	HEK293	Yes	GSE125240	(12)
14			brain	Yes	GSE125240	
15			kidney	Yes	GSE125240	
16			liver	Yes	GSE125240	
17	MAZTER-seq	fusion domain	HEK293T	Yes	GSE122961	(13)
18			ESC	Yes	GSE122961	
19	DART-seq		HEK293T	Yes	GSE125780	(14)
20	m6A-Label-seq	substrates alternation	HEK293T	Yes	GSE131316	(15)

*__Note:__ A total of 20 datasets were merged into 9 technique-specific epitranscriptomes. The nine epitranscriptome profiling techniques can be further classified into four categories. The datasets obtained from the same technique were merged to form a technique-specific epitranscriptome.

Supplementary Table S3 Data sources of 25 tissue-specific m^6A dataset

	GSA/GEO	Sources	Names	Ref.
1	CRP000736	Tissues	Adrenal gland	(16)
2			Brainstem	
3			Cerebellum	
4			Cerebrum	
5			Colon	
6			Heart	
7			Hypothalamus	
8			Liver	
9			Lung	
10			Muscle	
11			Prostate	
12			Rectum	
13			Skin	
14			Stomach	
16			Testis	
17			Thyroid gland	
18			Urinary bladder	
19	GSE120024		Islet	(17)
20	GSE93911		Endometrial	(18)
21	GSE122744		Kidney	(19)
22	GSE119168		Ovary	(20)
23	GSE132306	Cell lines	EndoC-βH1	(17)
24	GSE112970		RWPE-1	(21)
25	GSE113798		B-lymphocyte	(22)
26	GSE106122		Hematopoietic stem cells (HSCs)	(23)

Supplementary Table S4 m^1A sites obtained from different techniques

	Technique	Cell Line	Note	GEO	Ref
1	m^1A-seq	HEK293T	TRMT6/TRM61A overexpression	GSE97419	(24)
2	m^1A-seq	HEK293T	None	GSE97419	(24)
3	m1A-MAP	HEK293T	None	GSE102040	(25)
4	m1A-IP-seq	HEK293T	None	GSE123365	(26)
5	m1A-quant-seq	HEK293T	None	GSE123365	(26)

Supplementary Table S5 Performance of gridTX with different number of slices

Slices #	Accuracy	Recall	Precision	F1-score	MCC	AUC	AP
10	0.6385	0.8287	0.6003	0.6963	0.2995	0.6983	0.6681
20	0.6713	0.8264	0.6307	0.7154	0.3603	0.7325	0.6937
30	0.6948	0.8136	0.6574	0.7272	0.4010	0.7476	0.7073
40	0.6897	0.8158	0.6514	0.7244	0.3920	0.7540	0.7147
50	0.7059	0.8094	0.6706	0.7335	0.4210	0.7611	0.7194
60	0.7109	0.8155	0.6744	0.7383	0.4313	0.7644	0.7187
70	0.7111	0.8158	0.6745	0.7385	0.4317	0.7685	0.7285
80	0.7124	0.8026	0.6799	0.7362	0.4319	0.7726	0.7282
90	0.7114	0.8049	0.6782	0.7361	0.4305	0.7728	0.7293
100	0.7166	0.7984	0.6861	0.7380	0.4390	0.7734	0.7281

*[*]***Note**: The threshold used for Accuracy, Recall, Precision, F1-score, MCC is 0.5. AUC, the area under ROC curve; AP, average precision.

Supplementary Table S6 Performance of chunkTX with different number of chunks

Chunk #	Accuracy	Recall	Precision	F1-score	MCC	AUC	AP
25	0.7360	0.8011	0.7088	0.7521	0.4760	0.8006	0.7572
35	0.7371	0.8053	0.7087	0.7539	0.4787	0.8065	0.7643
45	0.7364	0.7970	0.7108	0.7514	0.4762	0.8058	0.7625

[*]**Note**: The threshold used for Accuracy, Recall, Precision, F1-score, MCC is 0.5. AUC, the area under ROC curve; AP, average precision.

Supplementary Table S7 Performance of m6A site predictors on 5'UTR sites

Model	Feature Type		Performance		
	Sequence	Geographic	MCC	AUC	AP
DeepPromise	One-hot	-	0.467±0.066	0.819±0.021	0.714±0.036
GepSe	One-hot	chunkTX (Longest)	0.521±0.053	0.841±0.023	0.751±0.053

***Note**: Each model was evaluated using 10-fold cross-validation. Only sites from the 5'-UTR were selected for evaluation. The positive-to-negative ratio is about 1:2. The results are provided in the form of an average ± standard deviation. MCC, Matthews correlation coefficient; AUC, the area under ROC curves; AP, average precision.

Supplementary Table S8 Performance of models on sites with different isoform ambiguities

Num. Isoforms	Features	Accuracy	F1-score	MCC	AUC	AP
1	Sequence	0.7938	0.7893	0.5895	0.8720	0.8368
	Sequence + one-hot	0.8219	0.8176	0.6455	0.8968	0.8634
	Sequence + chunkTX	0.8253	0.8229	0.6532	0.8993	0.8649
2 or 3	Sequence	0.7817	0.7955	0.5621	0.8571	0.8490
	Sequence + one-hot	0.8179	0.8294	0.6349	0.8901	0.8822
	Sequence + chunkTX	0.8239	0.8363	0.6472	0.8947	0.8833
Above 3	Sequence	0.7830	0.7876	0.5665	0.8627	0.8464
	Sequence + one-hot	0.8326	0.8324	0.6652	0.9022	0.8843
	Sequence + chunkTX	0.8390	0.8405	0.6781	0.9091	0.8925

*__Note__: The threshold used for Accuracy, F1-score, MCC is 0.5. AUC, the area under ROC curve; AP, average precision.

Supplementary Table S9 Pairwise comparison of nine techniques on all genes

	m^6A-seq	PA-m^6A-seq	miCLIP	m^6A-CLIP-seq	m^6A-REF-seq	MAZTER-seq	DART-seq	m^6ACE-seq	m^6A-Label-seq
m^6A-seq	10730	828	4937	3939	294	11	28	172	283
PA-m^6A-seq	7.72%	17161	4911	3388	448	170	294	265	277
miCLIP	46.01%	28.62%	64598	21691	2279	588	1264	1045	1528
m^6A-CLIP-seq	36.71%	19.74%	47.83%	45349	1656	350	868	849	1341
m^6A-REF-seq	2.74%	3.91%	19.88%	14.45%	11461	105	167	39	166
MAZTER-seq	0.10%	1.14%	3.93%	2.34%	0.92%	14944	318	2	15
DART-seq	0.35%	3.70%	15.89%	10.91%	2.10%	4.00%	7954	16	33
m^6ACE-seq	10.53%	16.22%	63.95%	51.96%	2.39%	0.12%	0.98%	1634	112
m^6A-Label-seq	7.85%	7.69%	42.41%	37.22%	4.61%	0.42%	0.92%	6.85%	3603

*__Note__: The diagonal elements show the total number of m^6A sites detected by a specific technique. The elements in the upper right triangle show the number of sites detected by two techniques simultaneously. The elements in the lower left triangle show the consistency score between two techniques, with 1 indicating perfect consistency and 0 indicating no consistency at all. The consistency score is calculated as follows. Let A and B represent the sets of m^6A sites uncovered by two different techniques, respectively, and $|A|$ represents the total number of sites contained within a set A. The consistency score of two techniques is calculated by: $s_{A,B} = |A \cap B|/\min(|A|,|B|)$. The introduction of the consistency score to compensate different stringency in bioinformatics analysis, with the assumption that, if a less stringent cut-off threshold is applied to the technique that generated smaller set of m^6A sites, more m^6A sites will be reported by this technique, and the percentage of m^6A sites that can be validated by the other technique remains the same for those newly identified m^6A sites.

Supplementary Table S10 Permutation analysis of the reproducibility between different techniques

Repeatability	Number of sites		Proportion		Estimated FDR
	Experiment	Permutation	Experiment	Permutation	
≥2	10038	4173	30.522%	9.882%	41.57%
≥3	2941	226	8.034%	0.526%	7.684%
≥4	584	7	1.445%	0.018%	1.199%

***Note:** Permutation was performed 100 times on the DRACH motifs of house-keeping genes only to minimize the impact of condition-specific gene expression.

Supplementary Table S11 Performance of m^{6}A site predictors on miCLIP2 non-DRACH sites

Model	Feature Type		Performance		
	Sequence	Geographic	MCC	AUC	AP
DeepPromise	One-hot	-	0.843±0.017	0.989±0.003	0.934±0.010
i-GepSe	One-hot	chunkTX (All)	0.880±0.017	0.994±0.001	0.961±0.008

***Note**: Positive data were collected from miCLIP2 and negative data were sampled from NNANN sites. Positive-to-negative ratio is 1:10. Strong sequence patterns were observed for positive sites: 37.8% of positive sites were from GGACG, and 15.0% were from GTACT. Each model was evaluated using 10-fold cross-validation. The results are provided in the form of an average ± standard deviation. MCC, Matthews correlation coefficient; AUC, the area under ROC curves; AP, average precision.

Supplementary Table S12 Performance evaluation of Geo2vec aided models on m^{1}A dataset

Model	Features	MCC	AUC	AP
CNN	Region type (251-nt)	0.276±0.063	0.696±0.030	0.712±0.029
CNN	gridTX (80 grids)	0.293±0.034	0.723±0.022	0.719±0.038
XGBoost	landmarkTX	0.308±0.024	0.724±0.012	0.736±0.012
CNN	chunkTX	0.347±0.034	0.748±0.018	0.764±0.018
DeepPromise	Sequence	0.374±0.059	0.750±0.031	0.737±0.037
DeepRiPe	Sequence + One-hot (251-nt)	0.400±0.095	0.782±0.034	0.791±0.037
GepSe	Sequence + chunkTX (Longest)	**0.527±0.063**	0.842±0.028	0.855±0.026
i-GepSe	Sequence + chunkTX (All)	0.521±0.026	**0.855±0.021**	**0.872±0.017**

***Note**: The dataset was constructed with a positive-negative ratio of 1:1. Each model was trained using 5-fold cross-validation. The threshold used for MCC is 0.5. Bold font indicates the best performance among the models. AUC, the area under ROC curve; AP, average precision.

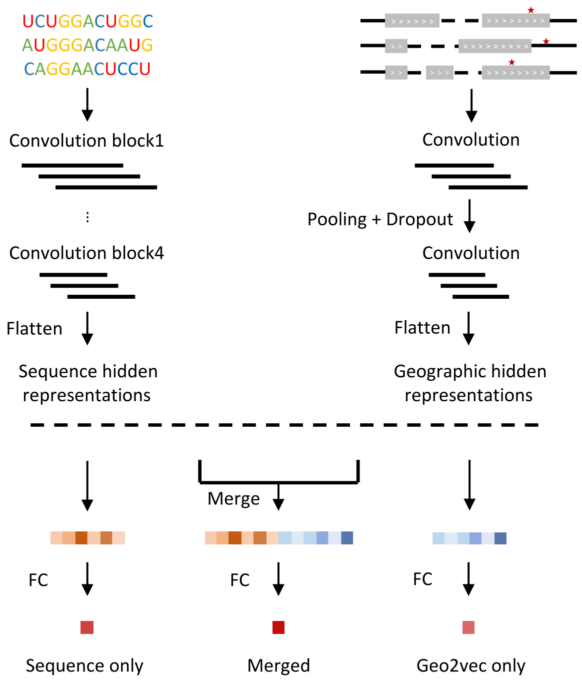

Model	DeepPromise	DeepRiPe	DeepRiPe	Multi-model DeepPromise
**Path**	Sequence	Sequence	Region	Geo2vec
**Number of CNN**	4	1	1	2
**Number of Kernels**	64/64/64/64	90	90	64/32
**Kernel Size**	5	7	7	5/3
**Pool Size**	2	4	10	2
**Pool Stride**	2	2	5	2

Supplementary Figure S1. Simplified graphical illustration of GepSe model. Sequence only, DeepPromise with four convolution blocks. Each block contains a convolutional layer, a max-pooling layer, and a dropout layer. Geo2vec only, 2-layer convolutional networks for geographic information-based modeling. Merged, the sequence hidden representations and geographic hidden representations are first merged together and then passed to fully connected layers for prediction. FC, fully connected layers. DeepRiPe and GepSe have only one max-pooling layer after the first convolutional layer.

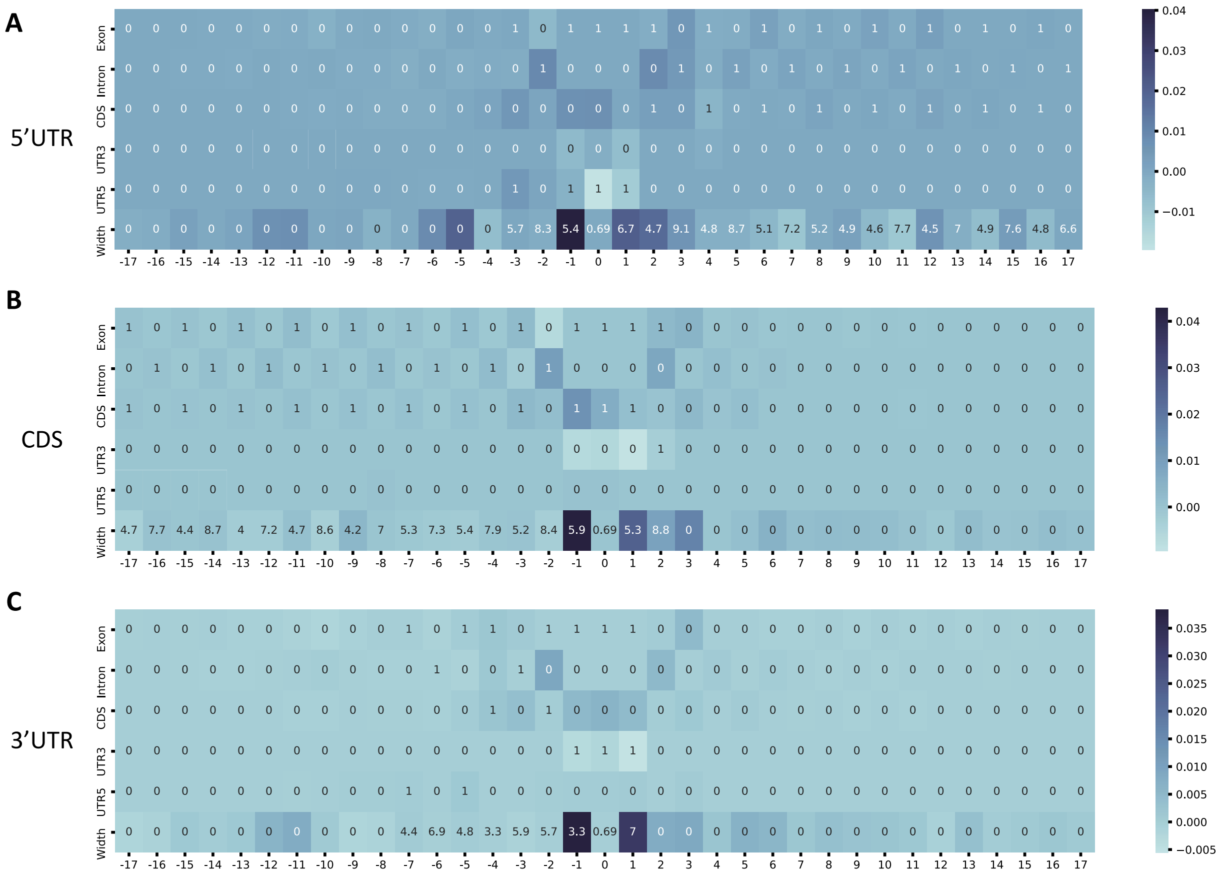

Supplementary Figure S2. Interpretation of chunkTX using deep SHAP. All training data were used as background, and the interpretation was conducted on each test data. The color bar indicates the contribution of each feature to the m^{6}A prediction. The higher the value, the greater the impact. The negative value means the opposite contribution to the prediction. The three heatmaps are selected from the predicted true positives with the highest predicted value in each category. The values on the heatmap are the original feature values. The width of the region was log-transformed. High positive impacts were observed on both -1_width and 1_width, indicating the importance of exon length. For CDS and 3'UTR, the zero-width of the chunk next to the right of the last 3'UTR shows positive impacts on m^{6}A prediction, indicating m^{6}A is enriched at the last exon.

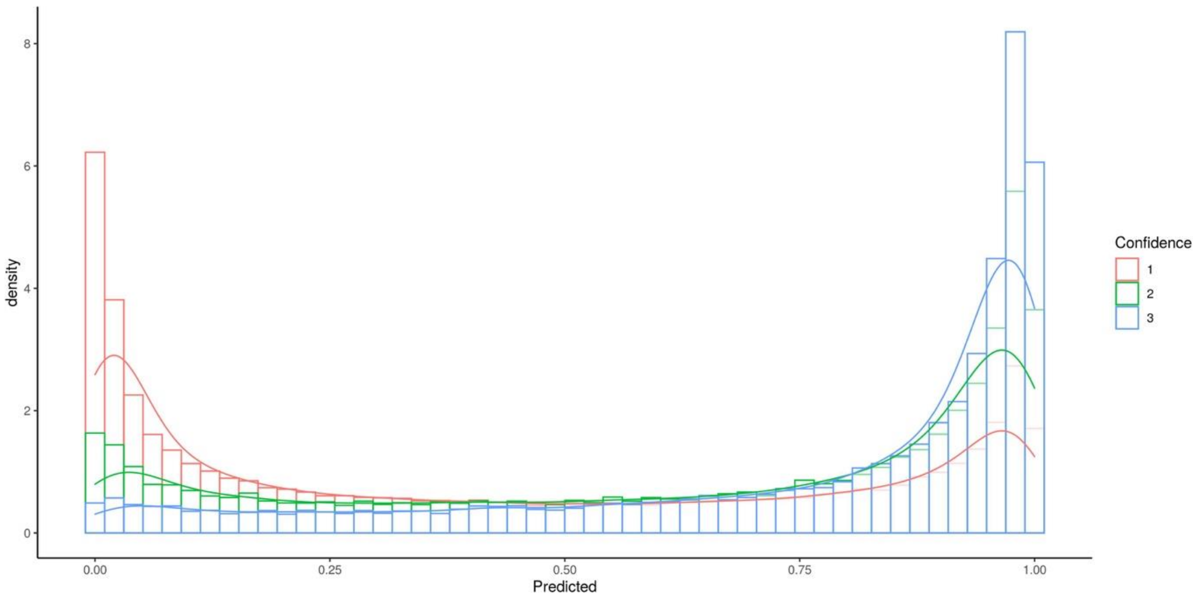

Supplementary Figure S3. Predicted scores of experimentally reported m^{6}A sites based on i-GepSe trained on sites detected by at least 4 techniques. Confidence 1, 2, and 3: m^{6}A sites supported by one experimental sample (n=83,694), two experimental samples (n=26,049), and three experimental samples (n=13,098), respectively. The higher the prediction score, the more confident the model is in identifying the site as methylated.

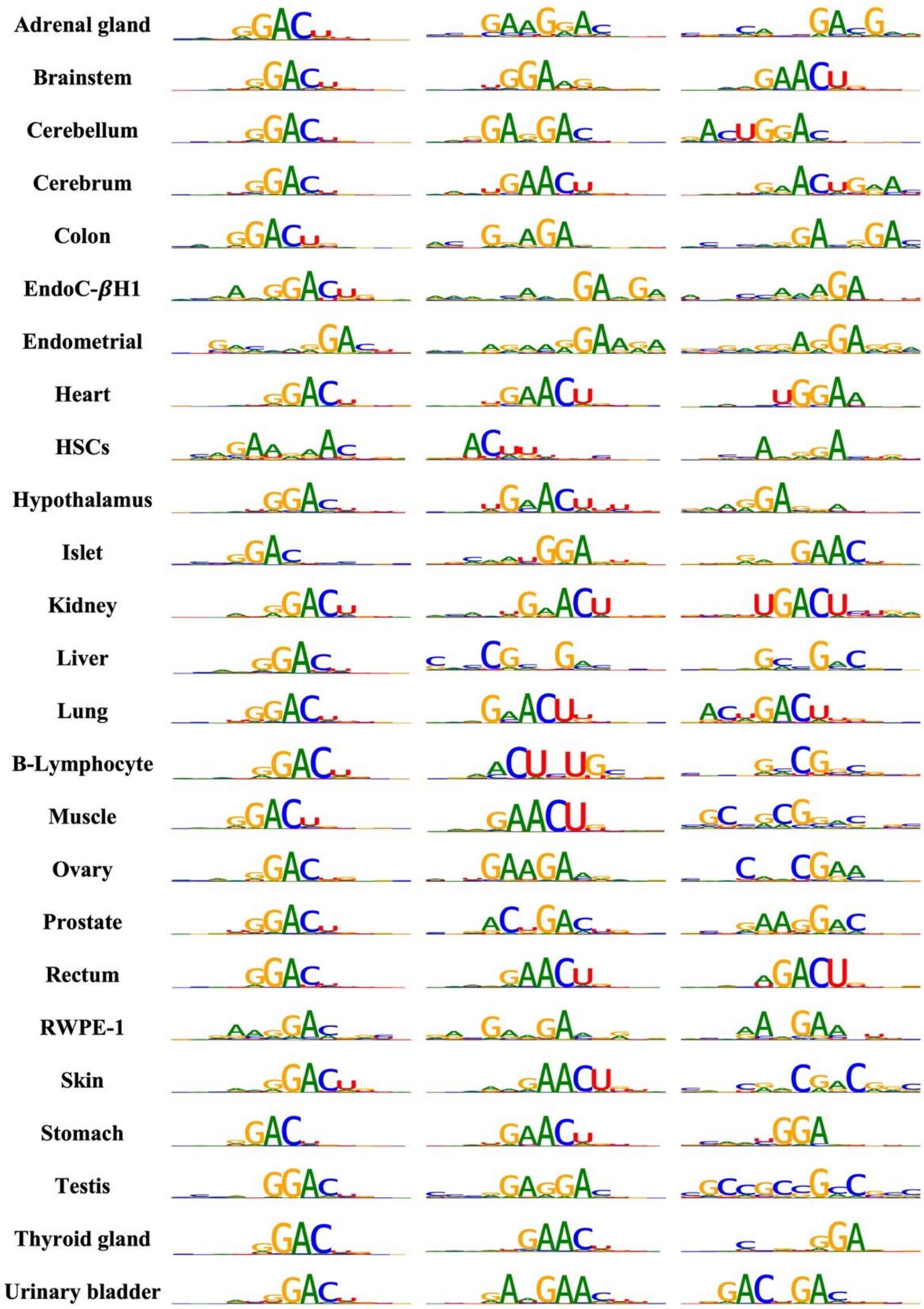

Supplementary Figure S4. Top m^{6}A motifs in 25 tissues/cells detected by ti-GepSe. Motif mining was performed using deep learning interpretation method Integrated Gradients and the motif discovery software tf-MoDISco.

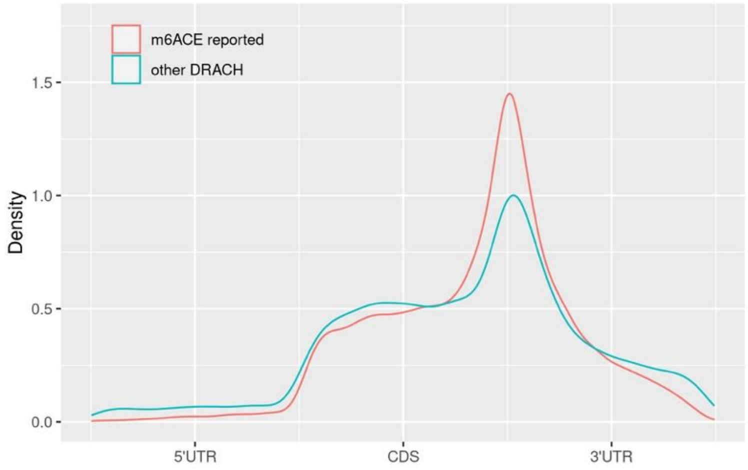

Supplementary Figure S5. The relative position distribution of sites that were predicted to be methylated from Nanopore sequencing data. m6ACE reported: the HEK293T m^{6}A sites collected from m6ACE-seq; other DRACH: all experimentally undetected DRACH motifs from the same transcript habouring m6ACE-seq sites.

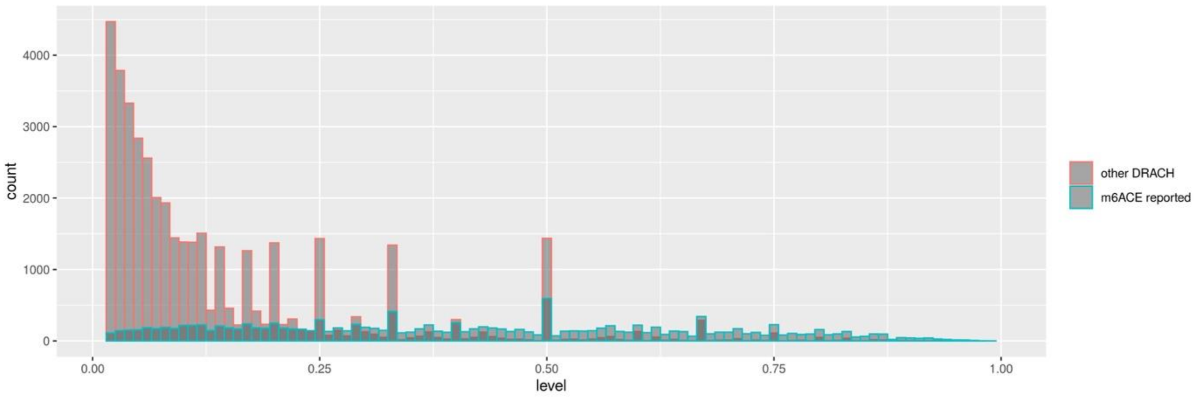

Supplementary Figure S6. Predicted methylation levels for sites with at least one read predicted to be methylated (level > 0). 190,282 experimentally undetected sites (~92% of total other DRACH) and 1,179 experimentally detected sites (~8% of total m6ACE reported) were predicted to have zero methylation levels. Level: the percentage of reads predicted to be methylated.

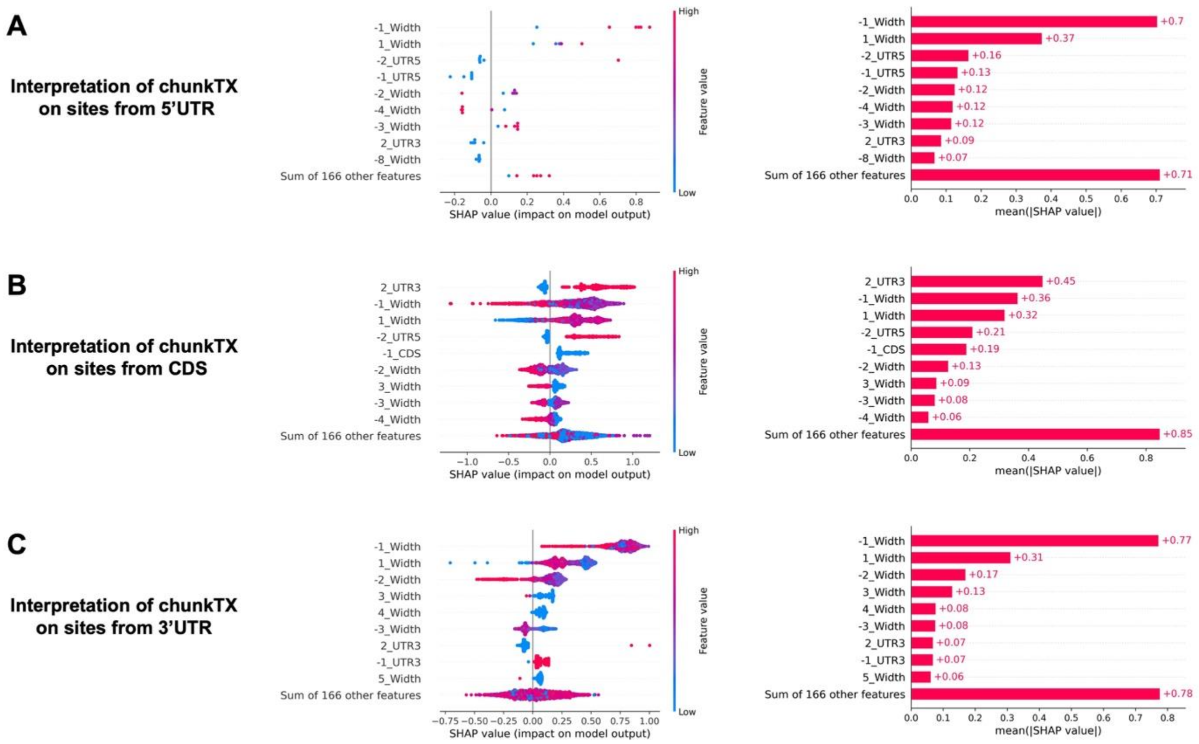

Supplementary Figure S7. Interpretation of chunkTX for non-DRACH motif sites from miCLIP2. We implemented Shapley additive explanations (SHAP) to obtain geographic explanation of Geo2vec on non-DRACH sites. Most sites (the number of points) are located in CDS and 3'UTR regions. For CDS and 3'UTR sites, "-1_Width" and "1_Width, the distance of the site to the two boundaries of the region where it is located, were consistently included in the Top 3 features, indicating the importance of its relative position in the exon and the width of that exon. Interestingly, "2_UTR3" and "-2_UTR5" were the Top 1 and Top 4 features for CDS site prediction, which provided some evidence that the non-DRACH motif site prefers CDS regions directly linked to the 3'UTR or 5'UTR. However, since sequence patterns dominated the prediction of non-DRACH motif sites (**Supplementary Table S11**), the interpretation of Geo2vec may not be strong enough to reflect all transcript preferences of non-DRACH motif sites.

REFERENCES

1. Schwartz, S., Mumbach, M.R., Jovanovic, M., Wang, T., Maciag, K., Bushkin, G.G., Mertins, P., Ter-Ovanesyan, D., Habib, N., Cacchiarelli, D. *et al.* (2014) Perturbation of m6A writers reveals two distinct classes of mRNA methylation at internal and 5' sites. *Cell reports*, **8**, 284-296.
2. Chen, K., Lu, Z., Wang, X., Fu, Y., Luo, G.Z., Liu, N., Han, D., Dominissini, D., Dai, Q., Pan, T. *et al.* (2015) High-resolution N(6) -methyladenosine (m(6) A) map using photo-crosslinking-assisted m(6) A sequencing. *Angewandte Chemie (International ed. in English)*, **54**, 1587-1590.
3. Ke, S., Alemu, E.A., Mertens, C., Gantman, E.C., Fak, J.J., Mele, A., Haripal, B., Zucker-Scharff, I., Moore, M.J., Park, C.Y. *et al.* (2015) A majority of m6A residues are in the last exons, allowing the potential for 3′ UTR regulation. *Genes and Development*, **29**, 2037--2053.
4. Ke, S., Pandya-Jones, A., Saito, Y., Fak, J.J., Vågbø, C.B., Geula, S., Hanna, J.H., Black, D.L., Darnell, J.E., Jr. and Darnell, R.B. (2017) m(6)A mRNA modifications are deposited in nascent pre-mRNA and are not required for splicing but do specify cytoplasmic turnover. *Genes & development*, **31**, 990-1006.
5. Linder, B., Grozhik, A.V., Olarerin-George, A.O., Meydan, C., Mason, C.E. and Jaffrey, S.R. (2015) Single-nucleotide-resolution mapping of m6A and m6Am throughout the transcriptome. *Nature methods*, **12**, 767-772.
6. Boulias, K., Toczydłowska-Socha, D., Hawley, B.R., Liberman, N., Takashima, K., Zaccara, S., Guez, T., Vasseur, J.J., Debart, F., Aravind, L. *et al.* (2019) Identification of the m(6)Am Methyltransferase PCIF1 Reveals the Location and Functions of m(6)Am in the Transcriptome. *Mol Cell*, **75**, 631-643.e638.
7. van Tran, N., Ernst, F.G.M., Hawley, B.R., Zorbas, C., Ulryck, N., Hackert, P., Bohnsack, K.E., Bohnsack, M.T., Jaffrey, S.R., Graille, M. *et al.* (2019) The human 18S rRNA m6A methyltransferase METTL5 is stabilized by TRMT112. *Nucleic Acids Res*, **47**, 7719-7733.
8. Meyer, K.D., Patil, D.P., Zhou, J., Zinoviev, A., Skabkin, M.A., Elemento, O., Pestova, T.V., Qian, S.B. and Jaffrey, S.R. (2015) 5' UTR m(6)A Promotes Cap-Independent Translation. *Cell*, **163**, 999-1010.
9. Vu, L.P., Pickering, B.F., Cheng, Y., Zaccara, S., Nguyen, D., Minuesa, G., Chou, T., Chow, A., Saletore, Y., MacKay, M. *et al.* (2017) The N(6)-methyladenosine (m(6)A)-forming enzyme METTL3 controls myeloid differentiation of normal hematopoietic and leukemia cells. *Nature medicine*, **23**, 1369-1376.
10. Huang, H., Weng, H., Zhou, K., Wu, T., Zhao, B.S., Sun, M., Chen, Z., Deng, X., Xiao, G., Auer, F. *et al.* (2019) Histone H3 trimethylation at lysine 36 guides m(6)A RNA modification co-transcriptionally. *Nature*, **567**, 414-419.
11. Koh, C.W.Q., Goh, Y.T. and Goh, W.S.S. (2019) Atlas of quantitative single-base-resolution N(6)-methyl-adenine methylomes. *Nat Commun*, **10**, 5636.
12. Zhang, Z., Chen, L.Q., Zhao, Y.L., Yang, C.G., Roundtree, I.A., Zhang, Z., Ren, J., Xie, W., He, C. and Luo, G.Z. (2019) Single-base mapping of m(6)A by an antibody-independent method. *Science advances*, **5**, eaax0250.
13. Garcia-Campos, M.A., Edelheit, S., Toth, U., Safra, M., Shachar, R., Viukov, S., Winkler, R., Nir, R., Lasman, L., Brandis, A. *et al.* (2019) Deciphering the "m(6)A Code" via Antibody-Independent Quantitative Profiling. *Cell*, **178**, 731-747.e716.

14. Meyer, K.D. (2019) DART-seq: an antibody-free method for global m(6)A detection. *Nature methods*, **16**, 1275-1280.
15. Shu, X., Cao, J., Cheng, M., Xiang, S., Gao, M., Li, T., Ying, X., Wang, F., Yue, Y., Lu, Z. *et al.* (2020) A metabolic labeling method detects m(6)A transcriptome-wide at single base resolution. *Nature chemical biology*, **16**, 887-895.
16. Liu, J.e., Li, K., Cai, J., Zhang, M., Zhang, X., Xiong, X., Meng, H., Xu, X., Huang, Z., Peng, J. *et al.* (2020) Landscape and Regulation of m6A and m6Am Methylome across Human and Mouse Tissues. *Molecular Cell*, **77**, 426--440.e426.
17. De Jesus, D.F., Zhang, Z., Kahraman, S., Brown, N.K., Chen, M., Hu, J., Gupta, M.K., He, C. and Kulkarni, R.N. (2019) m(6)A mRNA Methylation Regulates Human β-Cell Biology in Physiological States and in Type 2 Diabetes. *Nat Metab*, **1**, 765-774.
18. Liu, J., Eckert, M.A., Harada, B.T., Liu, S.M., Lu, Z., Yu, K., Tienda, S.M., Chryplewicz, A., Zhu, A.C., Yang, Y. *et al.* (2018) m 6 A mRNA methylation regulates AKT activity to promote the proliferation and tumorigenicity of endometrial cancer. *Nature Cell Biology*, **20**, 1074--1083.
19. Zhang, H., Shi, X., Huang, T., Zhao, X., Chen, W., Gu, N. and Zhang, R. (2020) Dynamic landscape and evolution of m6A methylation in human. *Nucleic Acids Research*, **48**, 6251--6264.
20. Zhang, Z., Zhan, Q., Eckert, M., Zhu, A. and Chryplewicz, A.a. (2019) RADAR: Differential analysis of MeRIP-seq data with a random effect model. *Genome Biology*, **20**.
21. Yang, F., Jin, H., Que, B., Chao, Y., Zhang, H., Ying, X., Zhou, Z., Yuan, Z., Su, J., Wu, B. *et al.* (2019) Dynamic m6A mRNA methylation reveals the role of METTL3-m6A-CDCP1 signaling axis in chemical carcinogenesis. *Oncogene*, **38**, 4755--4772.
22. Engel, M., Eggert, C., Kaplick, P.M., Eder, M., Rh, S., Tietze, L., Namendorf, C., Arloth, J., Weber, P., Rex-Haffner, M. *et al.* (2018) The Role of m6A/m-RNA Methylation in Stress Response Regulation. *Neuron*, **99**, 389.
23. Kuppers, D.A., Arora, S., Lim, Y., Lim, A.R., Carter, L.M., Corrin, P.D., Plaisier, C.L., Basom, R., Delrow, J.J. and Wang, S.a. (2019) N6-methyladenosine mRNA marking promotes selective translation of regulons required for human erythropoiesis. *Nature Communications*, **10**.
24. Safra, M., Sas-Chen, A., Nir, R., Winkler, R., Nachshon, A., Bar-Yaacov, D., Erlacher, M., Rossmanith, W., Stern-Ginossar, N. and Schwartz, S. (2017) The m1A landscape on cytosolic and mitochondrial mRNA at single-base resolution. *Nature*, **551**, 251-255.
25. Li, X., Xiong, X., Zhang, M., Wang, K., Chen, Y., Zhou, J., Mao, Y., Lv, J., Yi, D., Chen, X.W. *et al.* (2017) Base-Resolution Mapping Reveals Distinct m(1)A Methylome in Nuclear- and Mitochondrial-Encoded Transcripts. *Mol Cell*, **68**, 993-1005.e1009.
26. Zhou, H., Rauch, S., Dai, Q., Cui, X., Zhang, Z., Nachtergaele, S., Sepich, C., He, C. and Dickinson, B.C. (2019) Evolution of a reverse transcriptase to map N(1)-methyladenosine in human messenger RNA. *Nature methods*, **16**, 1281-1288.
